# Structure and dynamics of small-scale turbulence in vaporizing two-phase flows

**DOI:** 10.1038/s41598-021-94334-x

**Published:** 2021-07-27

**Authors:** Radouan Boukharfane, Aimad Er-raiy, Matteo Parsani, Nilanjan Chakraborty

**Affiliations:** 1 Mohamed VI Polytechnic University (UM6P), MSDA, 43150 Benguerir, Morocco; 2 Computer Electrical and Mathematical Science and Engineering Division (CEMSE), Extreme Computing Research Center (ECRC), King Abdullah University of Science and Technology (KAUST), Thuwal, 23955 Saudi Arabia; 3 School of Engineering, Newcastle University, Claremont Road, Newcastle-Upon-Tyne, NE1 7RU UK

**Keywords:** Engineering, Mechanical engineering

## Abstract

Improving our fundamental understanding of multiphase turbulent flows will be beneficial for analyses of a wide range of industrial and geophysical processes. Herein, we investigate the topology of the local flow in vaporizing forced homogeneous isotropic turbulent two-phase flows. The invariants of the velocity-gradient, rate-of-strain, rate-of-rotation tensors, and scalar gradient were computed and conditioned for different distances from the liquid–gas surface. A Schur decomposition of the velocity gradient tensor into a normal and non-normal parts was undertaken to supplement the classical double decomposition into rotation and strain tensors. Using direct numerical simulations results, we show that the joint probability density functions of the second and third invariants have classical shapes in all carrier-gas regions but gradually change as they approach the carrier-liquid interface. Near the carrier-liquid interface, the distributions of the invariants are remarkably similar to those found in the viscous sublayer of turbulent wall-bounded flows. Furthermore, the alignment of both vorticity and scalar gradient with the strain-rate field changes spatially such that its universal behaviour occurs far from the liquid–gas interface. We found also that the non-normal effects of the velocity gradient tensor play a crucial role in explaining the preferred alignment.

## Introduction

Two-phase gas–liquid turbulent flows featuring phase changes are encountered in many natural processes, such as blood flow and rain formation, and numerous industrial applications, such as porous insulation materials, igneous intrusion in geothermal reservoirs, airlift pumps, heat exchange, boilers and nuclear/chemical reactors. The effectiveness of such systems is heavily dependant on parameters such as volume and residence time of discrete phase, bubble rise velocity, bubble size or bubble size distribution, and bubble deformability, which directly influence the momentum, heat, and mass transfer. The inherent nonlinearity of such flows, together with the various challenges of conducting experimental measurements, makes computational approaches an indispensable tool for the design and analysis of two-phase turbulence and phase distribution phenomena in numerous technological and natural problems^1^.

Velocity gradients and scalar gradients are used to understand many important turbulence processes such as scalar mixing, cascade dynamics, material element deformation, evolution of coherent structures, intermittency, and other small-scale turbulence process^2^. Based on the local streamline topology classification methodology introduced by Perry & Chong^3^ and Chong et al.^4^, small-scale flow structures in incompressible flows can be categorized into two nodal and two focal topologies. This classification is crucial for analysing the different manifestations of coherent structures. In this regard, the first stage of turbulence modelling studies relatively simple and well-understood configurations. For example, in single-phase flows, direct numerical simulation (DNS) of homogeneous isotropic turbulence (HIT), which is an idealized state of turbulence, is used to study scalar mixing together with Kolmogorov’s theory of turbulent fluid motion. For a review of the dynamics of small-scale turbulence and several kinds of modelling approaches, the reader is referred to Meneveau^5^. Extending this configuration to two-phase flows can characterize important processes involving highly turbulent and convoluted interfaces, as well as the breakup and coalescence of liquid masses due to vortical and straining flow motions, and/or external forces, has been the subject of several recent DNS studies.

A few studies have concentrated on the interaction between liquid breakup and turbulence^6^. Recently, by virtue of the advancing computational resources, this topic has been investigated using accurate interface tracking methods^7^. Duret et al.^8^ studied vaporization using DNS of two-phase HIT with evaporation in the context of turbulent primary atomization and mixing. Using the same numerical parameters and physical properties, Bouali et al.^9^ studied the effect of vaporization on scalar dissipation rate (SDR) statistics using a passive scalar to represent evaporation and mixing processes in two-phase turbulent flows. Hasslberger et al.^10^ investigated the topological features of turbulent fluid flows in an atomizing liquid jet by the probability distribution of topology volume fractions and size distributions. Recently, the methodology of studying local topology in the context of multiphase flows has been applied to analyse flow structures in turbulence generated by rising bubbles^11^ and in decaying droplet-laden isotropic turbulence^12^.

The aim of the present work is to build upon these studies by characterizing flow structures in the vicinity of evaporation fronts in a forced HIT, using classical Hermitian/skew–Hermitian decompositions of the velocity gradient tensor and the recently proposed Schur decomposition approach introduced by Keylock^13,14^, which splits the velocity gradient tensor into normal (characterized by the eigenvalues) and a non-normal (characterizing the tensor asymmetries) contributions. * The main purpose of this study is to i) improve the fundamental understanding of the mechanisms of evaporating droplets and turbulence interaction, and the one of the most useful tools is the investigation of the local topology through the use of Schur and Cauchy decomposition and ii) provide insight regarding the limitations of the traditional Cauchy decomposition.* Insights obtained in this work are expected to be beneficial for the development of subgrid-scale models and data-driven modelling methodologies.

## Velocity gradient tensor decomposition

### Cauchy-Stokes decomposition

The general pattern of the three-dimensional streamlines in the immediate vicinity of an observer moving at any point within the flow field can be determined by considering at the nature of the eigenvalues, $\left( \lambda _1,\lambda _2,\lambda _3\right)$, of the velocity gradient tensor ${\mathcal {A}}_{ij}=\partial {\mathfrak {u}}_i/\partial x_j$ evaluated at that point, from which the symmetric strain-rate ${\varvec{\mathcal {S}}}^{\mathcal {A}}$ and skew-symmetric rotation-rate $\varvec{\Omega }^{{{\mathcal {A}}}}$ tensors are obtained as ${{\mathcal {S}}^{{{\mathcal {A}}}}_{ij}=\left( {\mathcal {A}}_{ij}+{\mathcal {A}}_{ji}\right) /2}$ and ${\Omega ^{{{\mathcal {A}}}}_{ij}=\left( {\mathcal {A}}_{ij}-{\mathcal {A}}_{ji}\right) /2}$, respectively. Ordering $\lambda _i$ as follows, $\lambda _1>\lambda _2>\lambda _3$, and knowing that for incompressible flows $\lambda _1+\lambda _2+\lambda _3=0$, the first eigenvector ($\mathbf{e }_1$) is associated with the extensive eigenvalue ($\lambda _1>0$), the third eigenvector ($\mathbf{e }_3$) with the compressive eigenvalue ($\lambda _3<0$) and the second, intermediate, eigenvector ($\mathbf{e }_2$) can be either compressive or extensive. The three invariants ${P^{{{\mathcal {A}}}}}$, ${Q^{{{\mathcal {A}}}}}$, and ${R^{{{\mathcal {A}}}}}$ of ${\mathcal {A}}_{ij}$ in incompressible flow are expressed as:

1 $$\begin{aligned} {P^{{{\mathcal {A}}}}=0,~ Q^{{{\mathcal {A}}}}=\frac{1}{2}\left( -{\mathcal {S}}^{{{\mathcal {A}}}}_{ij}{\mathcal {S}}^{{{\mathcal {A}}}}_{ij}+\Omega ^{{{\mathcal {A}}}}_{ij}\Omega ^{{{\mathcal {A}}}}_{ij}\right) ,~ R^{{{\mathcal {A}}}}=-\frac{1}{3}\left( {\mathcal {S}}^{{{\mathcal {A}}}}_{ij}{\mathcal {S}}^{{{\mathcal {A}}}}_{jk}{\mathcal {S}}^{{{\mathcal {A}}}}_{ki}+3\Omega ^{{{\mathcal {A}}}}_{ij}\Omega ^{{{\mathcal {A}}}}_{jk}{\mathcal {S}}^{{{\mathcal {A}}}}_{ki}\right) .} \end{aligned}$$

Similarly, the second and third invariants of ${\varvec{\mathcal {S}}}^{\mathcal {A}}$ are defined by its characteristic equation as follows:

2 $$\begin{aligned} {Q^{{{\mathcal {S}}}^{{{\mathcal {A}}}}}=-{\mathcal {S}}^{{{\mathcal {A}}}}_{ij}{\mathcal {S}}^{{{\mathcal {A}}}}_{ji}/2=-\frac{ \varepsilon   _0}{4\nu },} \end{aligned}$$

and

3 $$\begin{aligned} {R^{{{\mathcal {S}}}^{{{\mathcal {A}}}}}=-{\mathcal {S}}^{{{\mathcal {A}}}}_{ij}{\mathcal {S}}^{{{\mathcal {A}}}}_{jk}{\mathcal {S}}^{{{\mathcal {A}}}}_{ki}/3=-\lambda _1\lambda _2\lambda _3.} \end{aligned}$$

The only invariant of $\varvec{\Omega }^{{{\mathcal {A}}}}$ is:

4 $$\begin{aligned} {Q^{{\Omega }^{{{\mathcal {A}}}}}=\Omega ^{{{\mathcal {A}}}}_{ij}\Omega ^{{{\mathcal {A}}}}_{ij}/2=\omega ^{\mathcal {A}}_i\omega ^{\mathcal {A}}_i/4=Q^{{{\mathcal {A}}}}-Q^{{{\mathcal {S}}}^{{{\mathcal {A}}}}},} \end{aligned}$$

**Figure 1:**
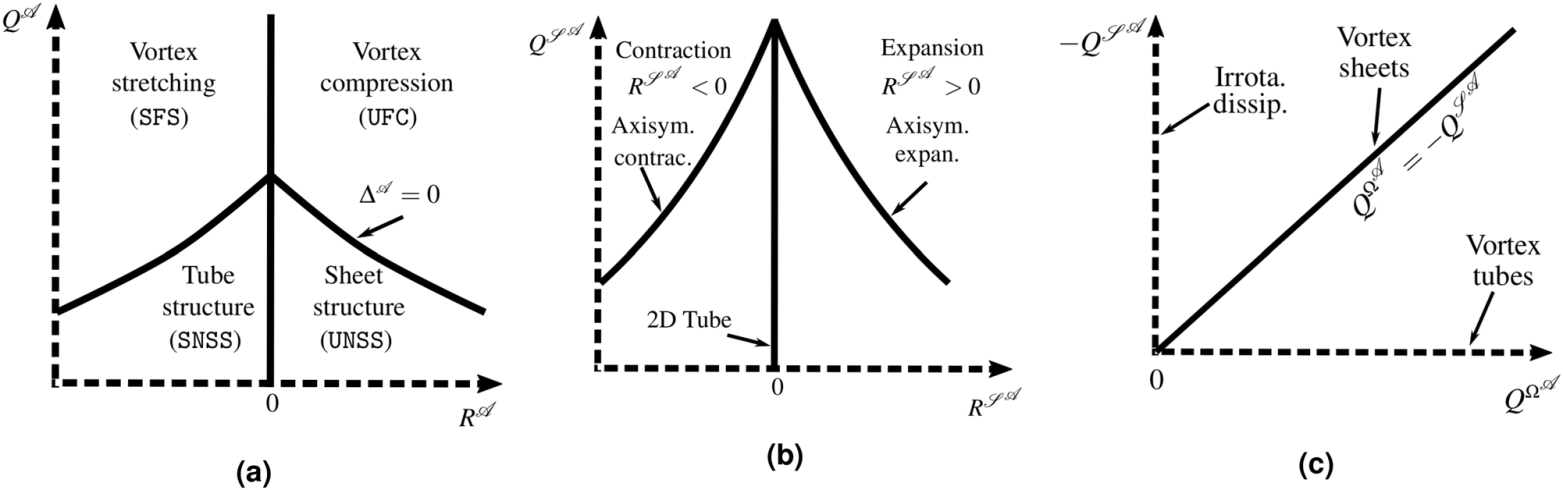
Sketch of the invariant maps of ${(Q^{{{\mathcal {A}}}},R^{{{\mathcal {A}}}})}$, ${(Q^{{{\mathcal {S}}}^{{{\mathcal {A}}}}},R^{{{\mathcal {S}}}^{{{\mathcal {A}}}}})}$ and ${(Q^{{\Omega }^{{{\mathcal {A}}}}},-Q^{{{\mathcal {S}}}^{{{\mathcal {A}}}}})}$ showing the physical and topological features related to each zone. In (**a**), SFS stands for stable-focus-stretching topology, UFS for unstable-focus-stretching topology, SNSS for stable node-saddle-saddle topology, and UNSS for unstable-node-saddle-saddle topology.

where ${\omega ^{\mathcal {A}}_i= \varepsilon   _{ijk}{\mathcal {A}}_{kj}}$ is the vorticity field. The invariants defined above are usually investigated in joint $\text {PDF}$s combining two invariants. The most common combinations consist on the maps of ${(Q^{{{\mathcal {A}}}},R^{{{\mathcal {A}}}})}$, ${(Q^{{{\mathcal {S}}}^{{{\mathcal {A}}}}},R^{{{\mathcal {S}}}^{{{\mathcal {A}}}}})}$ and ${(Q^{{\Omega }^{{{\mathcal {A}}}}},-Q^{{{\mathcal {S}}}^{{{\mathcal {A}}}}})}$. Figure 1 summarizes sketches of each of these maps, indicating the corresponding physical meaning of each particular location. The invariants ${R^{{{\mathcal {A}}}}}$ and ${Q^{{{\mathcal {A}}}}}$ are of great importance from a topological point of view, since the sign of the discriminant function ${\Delta ^{{{\varvec{\mathcal {A}}}}}=27R^{{{{\mathcal {A}}}},2}+4Q^{{{{\mathcal {A}}}},3}}$, which characterizes a different streamline flow pattern, is dependant on their magnitude. In the ${(Q^{{{\mathcal {A}}}},R^{{{\mathcal {A}}}})}$ space, we distinguish four non-degenerate topology types allowing us to infer the relationships between local flow topology and physical mechanisms,e.g., enstrophy production and dissipation rate production (see Fig. 1a). The ${(Q^{{{\mathcal {S}}}^{{{\mathcal {A}}}}},R^{{{\mathcal {S}}}^{{{\mathcal {A}}}}})}$ map is a useful tool for analysing the geometry of local strain in the fluid. In particular, since ${Q^{{{\mathcal {S}}}^{{{\mathcal {A}}}}}=- \varepsilon   _0/4\nu }$, large negative values of ${Q^{{{\mathcal {S}}}^{{{\mathcal {A}}}}}}$ are associated with regions of intense kinetic energy dissipation (see Fig. 1b). Finally, the ${(Q^{{\Omega }^{{{\mathcal {A}}}}},-Q^{{{\mathcal {S}}}^{{{\mathcal {A}}}}})}$ map is particularly useful to analyse topology associated with the dissipation of kinetic energy (see Fig. 1c). For a detailed description of each zone in the three maps, the reader is referred to Blackburn et al.^15^.

### Schur decomposition

The complex Schur decomposition applied to the velocity gradient tensor ${{\varvec{\mathcal {A}}} \in {\mathbb {C}}^{n \times n}}$, is given by

5 $${\varvec{\mathcal{A}}} ={{\mathfrak{U}} \mathcal{T} {\mathfrak{U}} }^{*},$$

where $*$ denotes the Hermitian of a complex matrix, $\mathfrak {\varvec{U}} \in {\mathbb {C}}^{3 \times 3}$ is a unitary matrix (i.e., $\mathfrak {\varvec{U}} \mathfrak {\varvec{U}}^*= \mathfrak {\varvec{I}}$), and ${{\varvec{\mathcal {T}}} \in {\mathbb {C}}^{3 \times 3}}$ is upper-triangular. Thus, the characteristic polynomials of ${{\varvec{\mathcal {A}}}}$ and ${{\varvec{\mathcal {T}}}}$ are the same and the roots of the latter are simply the diagonal elements of ${{\varvec{\mathcal {T}}}}$. This decomposition is valid for any complex or real matrix. The matrix ${{\varvec{\mathcal {T}}}}$ in (5) is given by:

6 $$\begin{aligned} {{\varvec{\mathcal {T}}}= \begin{pmatrix} \lambda _{11} &{} \Lambda _{12} &{} \Lambda _{13} \\ 0 &{} \lambda _{22} &{} \Lambda _{23} \\ 0 &{} 0 &{} \lambda _{33} \end{pmatrix} = \mathcal {\varvec{L}}+\varvec{\Lambda },} \end{aligned}$$

where $\mathcal {\varvec{L}}$ is a diagonal matrix whose elements correspond to the eigenvalues of ${{\varvec{\mathcal {A}}}}$, $\left( \lambda _1,\lambda _2,\lambda _3\right)$, and $\varvec{\Lambda }$ is an upper triangular matrix that represents the non-normal part of ${{\varvec{\mathcal {A}}}}$ (characterizing the matrix asymmetries). The velocity gradient tensor ${{\varvec{\mathcal {A}}}}$ can be decomposed into its normal, ${{\varvec{\mathcal {B}}}}$, and non-normal, ${{\varvec{\mathcal {C}}}}$, contributions^13^ as follows:

7 $$\begin{aligned} {{\varvec{\mathcal {A}}}={\varvec{\mathcal {B}}}+{\varvec{\mathcal {C}}}.} \end{aligned}$$

The linear algebra tool used to accomplish this is the complex Schur transform (5). To ensure the uniqueness of the decomposition of ${{\varvec{\mathcal {A}}}}$, the ordering of the eigenvalues of ${{\varvec{\mathcal {A}}}}$ is fixed in an increasing order, *i.e.*, $\lambda _{11}$ (resp. $\lambda _{33}$) represents the most negative (resp. the most positive) eigenvalue of ${{\varvec{\mathcal {A}}}}$. Applying transformation (5) to ${{\varvec{\mathcal {A}}}}$ obtain the following expressions:

8 $$\begin{aligned} {{\varvec{\mathcal {B}}}=\mathfrak {\varvec{U}}\mathcal {\varvec{L}}\mathfrak {\varvec{U}}^*,~{\varvec{\mathcal {C}}}=\mathfrak {\varvec{U}}\varvec{\Lambda }\mathfrak {\varvec{U}}^*.} \end{aligned}$$

Thus, we note that ${{\varvec{\mathcal {B}}}}$ acts locally and is related to dynamics driven by the eigenvalues of ${{\varvec{\mathcal {A}}}}$, whereas ${{\varvec{\mathcal {C}}}}$ is associated with vortical structures and non-local effects in the flow field. In order words, this decomposition explicitly gives i) information related to the eigenvalues of ${{\varvec{\mathcal {A}}}}$, which are associated with local dynamics, and ii) the non-symmetric structure in ${{\varvec{\mathcal {A}}}}$, which is induced by non-local effects. This information is not included in the conventional Cauchy-Stokes decomposition of ${{\varvec{\mathcal {A}}}}$. Note that non-normality could be measured by either ${\Vert {\varvec{\mathcal {A}}}{\varvec{\mathcal {A}}}^*-{\varvec{\mathcal {A}}}^*{\varvec{\mathcal {A}}}\Vert }$ or $\Vert \varvec{\Lambda }\Vert$, in which $\Vert \varvec{\phi }\Vert =\sqrt{\text {tr}\left( \varvec{\phi }\varvec{\phi }^*\right) }\equiv \sqrt{\sum _{i,j=1}^{3}|\phi _{ij}|^2}$ is the Frobenius norm of a generic quantity $\varvec{\phi }$. The following concepts and formula, which associate the Schur decomposition components with the invariants of ${{\varvec{\mathcal {A}}}}$, are detailed in Keylock^13^. As a second step, we consider the strain-rate and rotation-rate tensors of ${{\varvec{\mathcal {B}}}}$ and ${{\varvec{\mathcal {C}}}}$. The resulting expressions are:

9 $$\begin{aligned} {{\varvec{\mathcal {B}}}= {\varvec{\mathcal {S}}}^{{{\mathcal {B}}}}+ \varvec{\Omega }^{{{\mathcal {B}}}},~{\varvec{\mathcal {C}}}= {\varvec{\mathcal {S}}}^{{{\mathcal {C}}}}+ \varvec{\Omega }^{{{\mathcal {C}}}},} \end{aligned}$$

where

10 $$\begin{aligned} {{\varvec{\mathcal {S}}}^{{{\mathcal {B}}}}=\frac{1}{2}\left( {\varvec{\mathcal {B}}}+{\varvec{\mathcal {B}}}^*\right) ,~\varvec{\Omega }^{{{\mathcal {B}}}}=\frac{1}{2}\left( {\varvec{\mathcal {B}}}-{\varvec{\mathcal {B}}}^*\right) ,~ {\varvec{\mathcal {S}}}^{{{\mathcal {C}}}}=\frac{1}{2}\left( {\varvec{\mathcal {C}}}+{\varvec{\mathcal {C}}}^*\right) ,~\varvec{\Omega }^{{{\mathcal {C}}}}=\frac{1}{2}\left( {\varvec{\mathcal {C}}}-{\varvec{\mathcal {C}}}^*\right) .} \end{aligned}$$

In this case, the Frobenius norm of the strain and rotation tensors are as follows:

11 $$\begin{aligned} {\Vert {\varvec{\mathcal {S}}}^{\mathcal {A}}\Vert ^2 = \Vert {\varvec{\mathcal {S}}}^{{{\mathcal {B}}}}\Vert ^2+\Vert {\varvec{\mathcal {S}}}^{{{\mathcal {C}}}}\Vert ^2,~ \Vert \varvec{\Omega }^{{{\mathcal {A}}}}\Vert ^2~= \Vert \varvec{\Omega }^{{{\mathcal {B}}}}\Vert ^2+\Vert \varvec{\Omega }^{{{\mathcal {C}}}}\Vert ^2.} \end{aligned}$$

Since the eigenvalues of $${{\varvec{\mathcal {A}}}}$$ and $${{\varvec{\mathcal 
{B}}}}$$ are identical, the invariants of tensor $${{\varvec{\mathcal {A}}}}$$ are identical to those of $${{\varvec{\mathcal {B}}}}$$. Therefore, we obtain:

12 $$\begin{aligned} {{\left\{ \begin{array}{ll} P^{{\mathcal {B}}}=P^{{\mathcal {A}}}=P^{{\mathcal {S}}^{\mathcal {B}}},\\ Q^{{\mathcal {B}}}=Q^{{\mathcal {A}}}=Q^{{\mathcal {S}}^{\mathcal {B}}}+Q^{\Omega ^{\mathcal {B}}}= \frac{1}{2}\left( \Vert \varvec{\Omega }^{{{\mathcal {B}}}}\Vert -\Vert {\varvec{\mathcal {B}}}^{{\mathcal {B}}}\Vert \right) ,\\ R^{{\mathcal {B}}}=R^{{\mathcal {A}}}=Q^{{\mathcal {S}}^{\mathcal {B}}}-E^{{\mathfrak {p}}^{\mathcal {B}}}= -\text {det}\left( {\varvec{\mathcal {S}}}^{{{\mathcal {B}}}}\right) -\text {tr}\left( \left( \varvec{\Omega }^{{{\mathcal {B}}}}\right) ^2{\varvec{\mathcal {S}}}^{{{\mathcal {B}}}}\right) , \end{array}\right. }} \end{aligned}$$

and

13 $$\begin{aligned} P^{{\mathcal {C}}}=P^{{\mathcal {S}}^{\mathcal {C}}}=P^{\Omega ^{\mathcal {C}}}=0,~ Q^{{\mathcal {C}}}=Q^{{\mathcal {S}}^{\mathcal {C}}}+Q^{\Omega ^{\mathcal {C}}}=0,~ R^{{\mathcal {C}}}=Q^{{\mathcal {S}}^{\mathcal {C}}}-E^{{\mathfrak {p}}^{\mathcal {C}}}=0, \end{aligned}$$

where $E^{{\mathfrak {p}}^\phi }=\frac{1}{4}\omega _i{\mathcal {S}}^{\Phi }_{ij}\omega _j$ is the enstrophy production rate for the generic quantity $\varvec{\phi }$. Taking into consideration the above identities, we note that the dynamics of ${{\varvec{\mathcal {A}}}}$ are dictated only by the normal part, ${{\varvec{\mathcal {B}}}}$. The second and third invariants contain non-normal contributions when ${\Vert {\varvec{\mathcal {C}}}\Vert \ne 0}$. Two new terms arise in both the strain and enstrophy productions for the third invariant, *i.e.*,

$$\begin{aligned} {{\left\{ \begin{array}{ll} \det \left( {\varvec{\mathcal {S}}}^{\mathcal {A}}\right) =\det \left( {\varvec{\mathcal {S}}}^{{{\mathcal {B}}}}\right) -\text {tr}\left( \left( \varvec{\Omega }^{{{\mathcal {C}}}}\right) ^2{\varvec{\mathcal {S}}}^{{{\mathcal {B}}}}\right) +\det \left( {\varvec{\mathcal {S}}}^{{{\mathcal {C}}}}\right) ,\\ \text {tr}\left( \left( \varvec{\Omega }^{{{\mathcal {A}}}}\right) ^2{\varvec{\mathcal {S}}}^{\mathcal {A}}\right) =\text {tr}\left( \left( \varvec{\Omega }^{{{\mathcal {B}}}}\right) ^2{\varvec{\mathcal {S}}}^{{{\mathcal {B}}}}\right) +\text {tr}\left( \left( \varvec{\Omega }^{{{\mathcal {C}}}}\right) ^2{\varvec{\mathcal {S}}}^{{{\mathcal {B}}}}\right) -\det \left( {\varvec{\mathcal {S}}}^{{{\mathcal {C}}}}\right) . \end{array}\right. }} \end{aligned}$$

The strain production is the result of the sum of the normal strain production, the interaction production, and the non-normal production, while the enstrophy production is the sum of the normal enstrophy production, the interaction production, and the non-normal production.

## Methods

### Governing equations

In the following, the material properties of both phases, *i.e.*, density and viscosity, are assumed to be constant. Subscripts $\ell$ and ${\mathfrak {g}}$ denote the liquid and gas phases, respectively. In a computational cell, the local fraction of liquid volume is denoted $\alpha$. The governing equations of the two-component incompressible flow model consisting of the three-dimensional Navier–Stokes equations for the mixture velocity $\mathfrak {\varvec{u}}$ and pressure ${\mathfrak {p}}$ are as follows:

14 $$\begin{aligned} {\left\{ \begin{array}{ll} \nabla \cdot \mathfrak {\varvec{u}} = 0,\\ \partial _t \mathfrak {\varvec{u}}+\nabla \cdot \left( \mathfrak {\varvec{u}}\mathfrak {\varvec{u}}\right) =-\frac{1}{\rho }\left[ \nabla {\mathfrak {p}}+\nabla \cdot \left( 2 \mu \mathcal {\varvec{S}}\right) +\varvec{f}_{\sigma }\right] +\varvec{f}, \end{array}\right. } \end{aligned}$$

and the advection equation for volume fraction $\alpha$ is:

15 $$\begin{aligned} \partial _t\alpha +\nabla \cdot (\alpha \mathfrak {\varvec{u}})=0, \end{aligned}$$

where the mixture density $\rho =(1-\alpha )\rho _{{\mathfrak {g}}}+\alpha \rho _{\ell }$ and dynamic viscosity $\mu =(1-\alpha )\mu _{{\mathfrak {g}}}+\alpha \mu _{\ell }$ are computed from $\alpha$ and constant material parameters $\rho _{\ell }$, $\rho _{\mathfrak {g}}$, $\mu _{\ell }$, and $\mu _{\mathfrak {g}}$. $\mathcal {\varvec{S}}$ denotes the strain-rate tensor ($\mathcal {\varvec{S}}\equiv [\nabla \mathfrak {\varvec{u}}+\left( \nabla \mathfrak {\varvec{u}}\right) ^\top ]/2$), $\varvec{f}$ is the forcing term that prevents the turbulence from decaying, and $\varvec{f}_{\sigma }$ is the force per unit of volume due to surface tension calculated as $\varvec{f}_{\sigma }=\sigma \kappa \varvec{n}\delta (s)$, where $\sigma$ and $\kappa$ are the surface tension coefficient and the radius of curvature of the interface between the two fluids (*i.e.*, the droplet and the surrounding fluid), $\varvec{n}$ is the unit vector normal to the interface and directed towards the fluid, with respect to which the interface is concave (droplet), and $\delta$ is the Dirac $\delta$-function required to impose $\varvec{f}_{\sigma }$ only at the interface between the two fluids, and *s* is a normal coordinate centred at the interface, such that $s=0$ at the interface. To take into account the influence of passive vaporization on the concentration fields in the gas phase, an additional equation describing the evolution of a representative scalar $\varpi$ of a passive evaporation process with a low temperature level at the interface is set to be equal to the normalized vapour mass fraction, *i.e.*, $\varpi =\Upupsilon _v/\Upupsilon _{\text {vs}}$, where $\Upupsilon _v$ denotes the vapour mass fraction and $\Upupsilon _{\text {vs}}$ is the saturation value of that fraction based on the Clausius-Clapeyron equation. The scalar $\varpi$ is set to one at the interface following the methodology described in Duret et al.^16^; it then evolves in the gas phase by means of convection and diffusion as follows:

16 $$\begin{aligned} \partial _t\varpi +\nabla \cdot \left( \mathfrak {\varvec{u}}\varpi \right) =\nabla \cdot \left( {\mathcal {D}}\nabla \varpi \right) , \end{aligned}$$

where diffusion coefficient ${\mathcal {D}}$ is set to ${\mathcal {D}}=\mu _{{\mathfrak {g}}}/\rho _{{\mathfrak {g}}}$, corresponding to a unity Schmidt number. At the initialization of evaporation in the simulation, $\varpi$ is set to zero in the gas and unity at the interface and liquid. The scalar equation is solved for the entire domain. In the liquid, $\varpi$ is fixed at unity during the simulation.

### Numerical methods

The above governing equations (14) are solved based on the kernel of the two-component incompressible flows solver Aphrós. For a detailed description of the numerical algorithm used, readers are referred to Karnakov et al.^17^. The equations are discretized on a uniform Cartesian mesh using a finite volume method based on Chorin’s projection for pressure coupling^18^ and the Bell–Colella–Glaz scheme^19^. The Poisson equation is iteratively solved using the Hypre library^20^. To transport the interface, the solver uses the volume-of-fluid method VOF/PLIC with piecewise linear reconstruction^21^. In the VOF/PLIC method, the normals are estimated using the mixed Youngs-centred scheme, which is a hybridization of Youngs’ scheme and the height functions, combining the advantages of both methodologies. To compute the distance from the interface, the marching cubes algorithm^22^ is used to estimate the signed distance function ${\Phi }$, which measures the shortest distance to the liquid-gas interface, such that ${\Phi }=0$ at the iterface, ${\Phi }>0$ indicates liquid region and ${\Phi }<0$ indicates gas region.

To sustain the turbulent kinetic energy of the velocity field at a desired level during the numerical simulation, the control-based linear forcing approach of Bassenne et al.^23^, which was initially developed for the gas phase, is here extended here for the liquid-gas mixtures. This approach is used to achieve statistically steady target values of kinetic energy and energy dissipation rate after several large-eddy turn over times. The method of Bassenne et al.^23^ consists of introducing low-pass filtering to the flow as follows:

17 $$\begin{aligned} \varvec{f} =\frac{ \varepsilon   -{\mathcal {G}}\left( {\mathcal {K}}-{\mathcal {K}}_{\infty }\right) \tau _{\infty }}{\langle \mathfrak {\varvec{u}}\cdot \widetilde{\mathfrak {\varvec{u}}}\rangle }\widetilde{\mathfrak {\varvec{u}}} \end{aligned}$$

where ${\mathcal {K}}=\langle {\mathfrak {u}}_i{\mathfrak {u}}_i \rangle /2$ and $\varepsilon   =\langle \frac{\mu }{\rho }\frac{\partial {\mathfrak {u}}_i}{\partial x_j}\frac{{\mathfrak {u}}_i}{\partial x_j}\rangle$ are turbulent kinetic energy and the energy dissipation rate, respectively. ${\mathcal {K}}_{\infty }$ and $\varepsilon   _{\infty }$ denote their corresponding steady mean values, $\tau _{\infty }$ is the integral time based on ${\mathcal {K}}_{\infty }$ and $\varepsilon   _{\infty }$ as ${\mathcal {K}}_{\infty }/ \varepsilon   _{\infty }$, and ${\mathcal {G}}$ denotes the feedback controller gain. $\langle \cdot \rangle$ refers to the volumetric averaging operator, and low-pass filtering operation is represented using $\widetilde{\varvec{\cdot }}$.

### Numerical set-up

We initialized the flow with sufficiently enough liquid structures (more precisely, eight droplets were confined into a periodic domain whose sum is equal to the prescribed liquid volume fraction as in Duret et al.^16^). Subsequently. we applied the forcing procedure to reach a given level of turbulence intensity. Upon obtaining a statistically-converged field, the passive evaporation process was activated. This strategy ensures that the flow almost reaches equilibrium between kinetic energy and surface tension/viscous forces without generating either i) violent small-scale atomization that is difficult to treat numerically or ii) unphysical shear stress across the surface. The shortcomings of this approach are that i) it is highly time consuming, and ii) it is constrained by the moderate values of Reynolds numbers lower than those achievable for single phase flows. The setup under investigation is characterised by several key dimensionless parameters. Herein, all these parameters were considered when the gas reached a $40\%$ saturated state. The physical parameters used are similar to those in previous work^8,24^. The main difference between previous approaches and that used in the present study is the value of the forcing kinetic energy (doubled in this work to ${\mathcal {K}}_{\infty }=7.2~\text {m}^2/\text {s}^2$). Moreover, the forcing energy dissipation rate is set to $\varepsilon   _{\infty }=0.52\times 10^{6}~\text {m}^2/\text {s}^3$. The computational domain has a size $L^3$, with a uniform Cartesian grid featuring $512^3$ points. This resolution is also twice as large as that used in previous works, since the focus of the present analysis is to analyse small-scale features. The initial density and dynamic viscosity ratios are set to 30, leading to the same kinematic viscosity in both the liquid and gas phases. Surface density $\sigma$ is set to 0.0135 $\text {N}.\text {m}^{-1}$ and the box is filled with $\phi _{\ell }=10\%$. For both the liquid and gas phases, the Weber numbers can be defined in several ways. In this study, similarly to Dodd & Ferrante^25^, the numbers are defined based on velocity fluctuations, thus ${\mathcal {W}}{\mathfrak {e}}=\rho {\mathcal {K}}_{\infty } L/\sigma$. The Reynolds number is defined as $\text {Re}=\rho \sqrt{{\mathcal {K}}_{\infty }}L/\mu$ and the liquid Ohnesorge number as ${\mathcal {O}}{\mathfrak {h}}_{\ell }=\sqrt{{\mathcal {W}}{\mathfrak {e}}_{\ell }}/\text {Re}_{\ell }$. Another relevant non-dimensional parameter, which classically characterizes single-phase homogeneous isotropic turbulence, is the Taylor microscale Reynolds number $\text {Re}_{\lambda }$, which is defined as follows $\text {Re}_{\lambda }=2{\mathcal {K}}_{\infty }\sqrt{5\rho _{{\mathfrak {g}}}/3\mu _{{\mathfrak {g}}} \varepsilon   _0}$, where $\lambda =\sqrt{10\mu _{{\mathfrak {g}}}{\mathcal {K}}_{\infty }/\rho _{{\mathfrak {g}}} \varepsilon   _{\infty }}$ is the Taylor microscale. This simple configuration is representative of liquid–gas flows that may be encountered in application such injection and atomization^8^. The Kolmogorov length scale is defined as $\eta _{{\mathfrak {g}}}=\left( \nu _{{\mathfrak {g}}}^3/ \varepsilon   _{\infty }\right) ^{1/4}$. The resolution parameter $\Delta x/\eta _{{\mathfrak {g}}}$ is 1.78, where $\Delta x$ is the uniform grid spacing in each coordinate direction. This estimation ensures that small scale features are well resolved in the present simulation^26^. The corresponding set of fluid parameters is summarized in Table 1.

**Table 1:** Summary of properties of the direct numerical simulations.

$\rho _{\ell }/\rho _{{\mathfrak {g}}}$	$\mu _{\ell }/\mu _{{\mathfrak {g}}}$	$\sigma$ $[\text {N}.\text {m}^{-1}]$	L [m]	$\phi _{\ell }$	${\mathcal {W}}{\mathfrak {e}}_{{\mathfrak {g}}}$	${\mathcal {W}}{\mathfrak {e}}_{\ell }$	$\text {Re}_{\ell }$	$\text {Re}_{\lambda }$	${\mathcal {O}}{\mathfrak {h}}_{\ell }$	$\Delta x/\eta _{{\mathfrak {g}}}$	$\lambda /\eta _{{\mathfrak {g}}}$
30	30	0.0135	$1.5\times 10^{-4}$	0.10	2.0	60	620	31.25	0.0125	1.78	11.00

### Assessment of mesh convergence

Unlike the single phase flow, there is no theoretical framework to define the smallest length scale in turbulent liquid-gas flows. Consequently, to ensure that the turbulence is deemed developed and realistic, only a mesh convergence study can be performed. In this study, the assessment of grid convergence is evaluated on the basis of four metrics *which are based, respectively, on the: (i) total surface area per unit volume , (ii) temporal evolution of the normalized vapour mass fraction, (iii) velocity powerspectra and (iv) norm of the strain rate tensor*.

**Figure 2:**
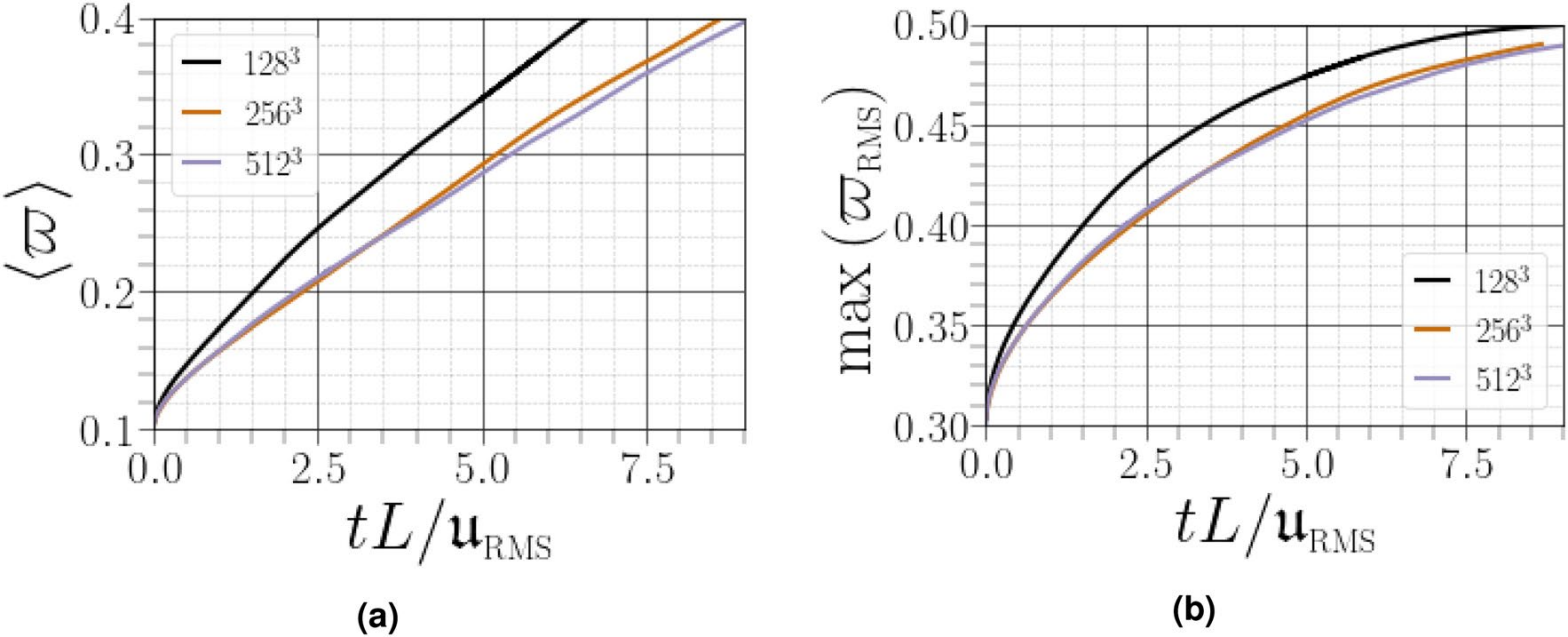
Temporal evolution of (**a**) the normalized mean vapour concentration and (**b**) the maximum of the scalar RMS of $\varpi$ for three levels of mesh resolution.

**Figure 3:**
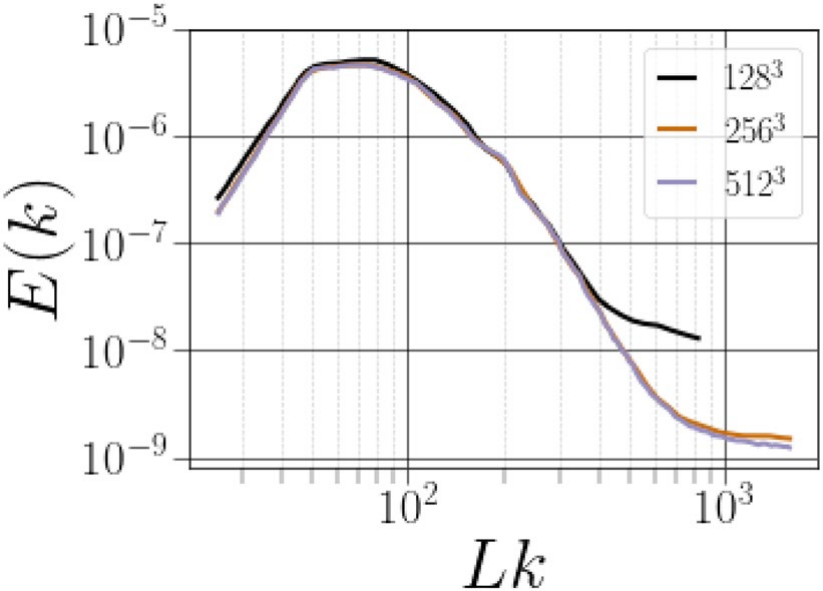
Energy spectra of the velocity field at the three grid resolutions.

* The first metric, is related to the mean liquid–gas surface density that determines the total amount of surface area divided by the simulation volume, denoted as* ${\overline{\Sigma }}$^24^ *(see Table* 2*). We noted that this metric quickly converged at higher resolutions, by 1% less when the mesh resolution of* $256^3$ *was modified to* $512^3$.* The second metric can describe the mixing and evaporation processes, which are estimated from the temporal evolution of the normalized vapour mass fraction* $\varpi$. *The temporal evolution of the mean scalar value* $\langle \varpi \rangle$ *and maximum value of the scalar RMS of* $\varpi$ *up to the time required for the gas to reach 40% of its saturation state is reported on Fig.* 2. *The high-resolution case exhibits a similar evolution of* $\langle \varpi \rangle$ *and* $\max \left( \varpi _{\text {RMS}}\right)$ *as in the lower-resolutions cases. The cases* $256^3$ *and* $512^3$ *exhibit small discrepancies, indicating, therefore, that the small-scale effects are resolved on the higher resolution mesh. A grid convergence based on the velocity spectra were performed, as shown in Fig.* 3. *A large range of the spectra remains unchanged between the three grid resolutions. However, the power spectra of* $256^3$ *resolution and* $512^3$ *resolution overlap in almost all scale ranges, which indicates the convergence of velocity power spectra under this present grid refinement. The last criteria is the norm of the strain rate tensor* $\langle {\mathcal {S}}_{ij}^{{\mathcal {A}}}{\mathcal {S}}_{ij}^{{\mathcal {A}}}\rangle$, *which can be considered as a good candidate to assess the small-scale features of the velocity field. This metrics converges to a plateau when the resolution reaches* $256^3$ *(see Table* 3). *Therefore, a resolution of* $512^3$ *could be considered as being fully appropriate for the present study.*

**Table 2:** Mesh convergence for the liquid–gas surface density.

Mesh resolution	$128^3$	$256^3$	$512^3$
$10^{-4}\times {\overline{\Sigma }}~[\text {m}^{-1}]$	2.2015	2.1731	2.1578

**Table 3:** Mesh convergence for the norm of the strain rate tensor.

Mesh resolution	$128^3$	$256^3$	$512^3$
$10^{-5}\times \sqrt{\langle {\mathcal {S}}_{ij}^{{\mathcal {A}}}{\mathcal {S}}_{ij}^{{\mathcal {A}}}\rangle }~[\text {s}^{-1}]$	5.1375	5.1498	5.1506

The invariants of the velocity-gradient tensor and the scalar $\varpi$ in the carrier fluid were analysed as the droplet interface approached. The second-order central difference is used to estimate all the gradients at every point in the flow, except near the liquid-gas interface, where the central difference stencil would lead to mixing between the liquid and gas velocities. In that scenario, the second-order one-sided scheme approximation was used. To discriminate the possible influence of the liquid-gas interface on both the velocity and scalar fields topologies, three layers were considered based on their distance from the interface as follows: $-2.8<{\Phi }/\eta _{{\mathfrak {g}}}<0$ (near the droplets surface), $-7<{\Phi }/\eta _{{\mathfrak {g}}}<-2.8$ (intermediate region between the carrier-fluid and the droplet) and ${\Phi }/\eta _{{\mathfrak {g}}}<-7$ (far from the droplets). These regions were labelled as ${\Phi }^{+}_c$ (c for close), ${\Phi }^{+}_i$ (i for intermediate) and ${\Phi }^{+}_f$ (f for far), respectively. We used the Kolmogorov length scale as the viscous scale reference quantity for normalizing distances from the interface. Dodd & Jofre^12^ used a viscous length scale based on the velocity and length scales near the droplet surface in their decaying isotropic turbulence laden with droplets of $\delta _{{\mathfrak {g}}}=\nu _{{\mathfrak {g}}}\sqrt{\rho _{{\mathfrak {g}}}/\tau _{{\mathfrak {g}}}}$, where $\tau _{{\mathfrak {g}}}=\mu _{{\mathfrak {g}}}\sqrt{4 \varepsilon   _0/15\nu _{{\mathfrak {g}}}}$. The relationship between $\eta _{{\mathfrak {g}}}$ and $\delta _{{\mathfrak {g}}}$ is $\delta _{{\mathfrak {g}}}=(15/4)^{1/4}\eta _{{\mathfrak {g}}}\approx 1.39\eta _{{\mathfrak {g}}}$. Therefore, the near surface to the droplet is twice the viscous length scale. The occupancy of near the droplets surface grid points (expressed as a percentage of realizations) is almost $6\%$, while the intermediate and far regions occupy 10% and 74%, respectively.

**Figure 4:**
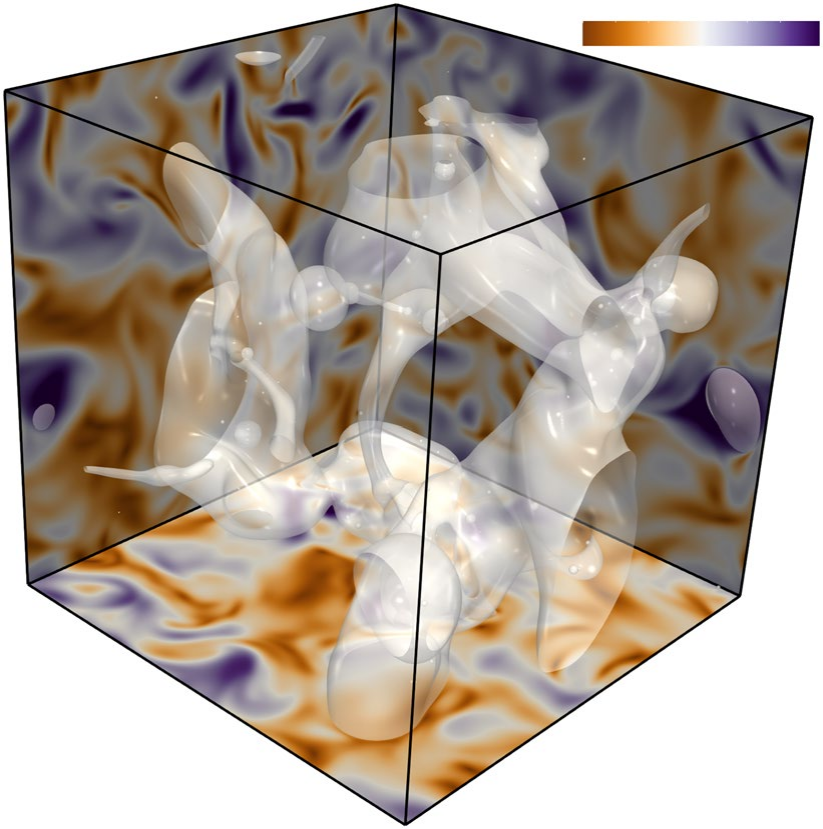
Instantaneous field of velocity magnitude with interface visualization (white).

Typical instantaneous snapshot of the liquid–gas turbulence flowfield is shown in Fig. 4 for the time at which the gas reaches 40% of the saturation state. Despite having a relatively small Weber number, the carrier-liquid interface undergoes complex turbulent breakup and appears highly wrinkled and tortuous, comprising elongated ligaments, drops, bag-like structures and bubbles, although the liquid was laminar at the initialization of the simulation.

## Results and discussion

### Invariants of the velocity gradient, rate-of-strain, and rate-of-rotation tensors considering distance from the interface

**Figure 5:**
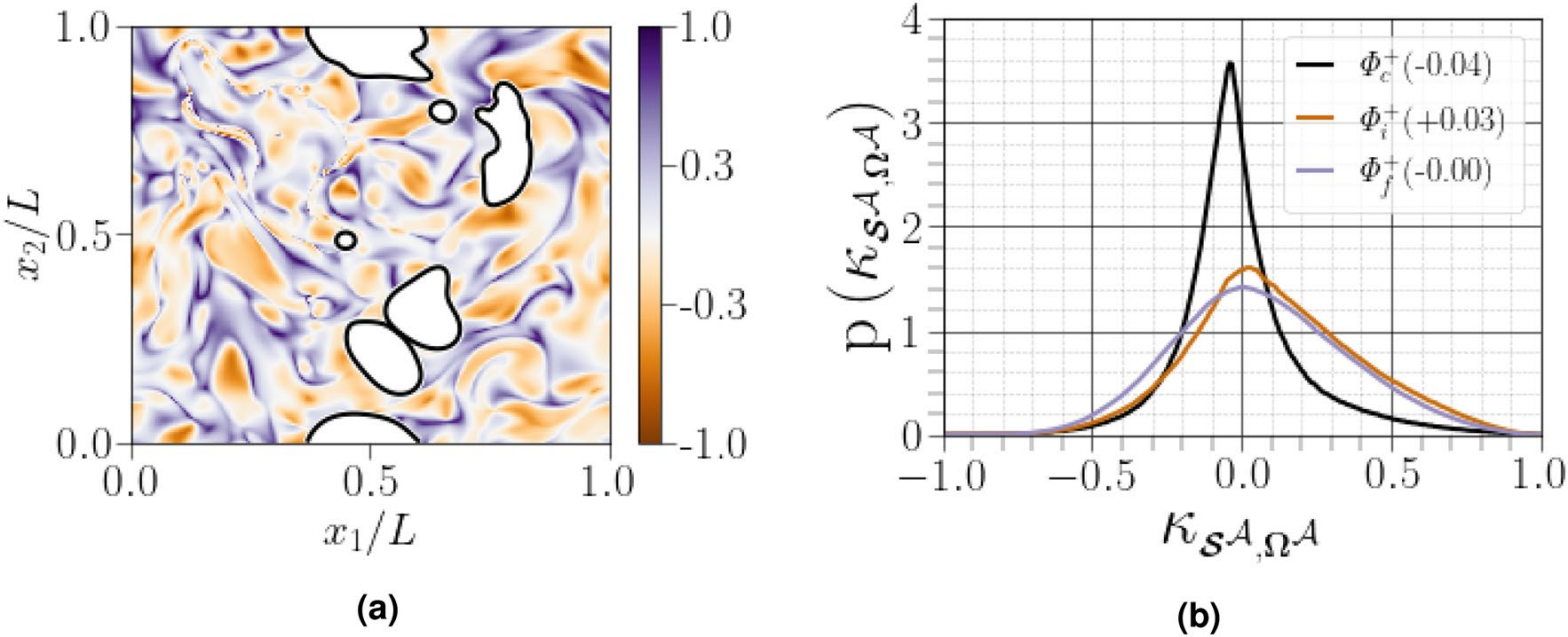
(**a**) PDF of the flow topology parameter ${\kappa _{\scriptscriptstyle {\varvec{\mathcal {S}}}^{\mathcal {A}},\varvec{\Omega }^{{{\mathcal {A}}}}}}$ in $x_3=L/2$ slice, where white spots correspond to the liquid phase interface, (**b**) PDF curves for ${\kappa _{\scriptscriptstyle {\varvec{\mathcal {S}}}^{\mathcal {A}},\varvec{\Omega }^{{{\mathcal {A}}}}}}$, the normalized difference in the Frobenius norms for ${\varvec{\mathcal {S}}}^{\mathcal {A}}$ and $\varvec{\Omega }^{{{\mathcal {A}}}}$ conditioned on different distances from the interface. Figures in parentheses indicate the mode of the corresponding PDF curve.

In this section, we study the dynamics, topology, and geometry of the gaseous fluid using the invariants of the velocity gradient, rate-of-strain, and rate-of-rotation tensors. All joint PDFs presented in this work cover $95\%$ of events observed in the flow at each distance from the liquid–gas surface. Firstly, we topologically classified the local flow regions using the standardized difference between ${\varvec{\mathcal {S}}}^{\mathcal {A}}$ and $\varvec{\Omega }^{{{\mathcal {A}}}}$ as proposed by^27^ ${\kappa _{\scriptscriptstyle {\varvec{\mathcal {S}}}^{\mathcal {A}},\varvec{\Omega }^{{{\mathcal {A}}}}}=(\Vert {\varvec{\mathcal {S}}}^{\mathcal {A}}\Vert -\Vert \varvec{\Omega }^{{{\mathcal {A}}}}\Vert )/(\Vert {\varvec{\mathcal {S}}}^{\mathcal {A}}\Vert +\Vert \varvec{\Omega }^{{{\mathcal {A}}}}\Vert )}$. Values of ${\kappa _{\scriptscriptstyle {\varvec{\mathcal {S}}}^{\mathcal {A}},\varvec{\Omega }^{{{\mathcal {A}}}}}=-1}$, ${\kappa _{\scriptscriptstyle {\varvec{\mathcal {S}}}^{\mathcal {A}},\varvec{\Omega }^{{{\mathcal {A}}}}}=0}$ and ${\kappa _{\scriptscriptstyle {\varvec{\mathcal {S}}}^{\mathcal {A}},\varvec{\Omega }^{{{\mathcal {A}}}}}=1}$ correspond to pure rotational flow, pure shear flow and pure elongational flow, respectively. If ${1/3<\kappa _{\scriptscriptstyle {\varvec{\mathcal {S}}}^{\mathcal {A}},\varvec{\Omega }^{{{\mathcal {A}}}}}<1}$, the region is dominated by strain, if ${-1<\kappa _{\scriptscriptstyle {\varvec{\mathcal {S}}}^{\mathcal {A}},\varvec{\Omega }^{{{\mathcal {A}}}}}<-1/3}$, the region is dominated by eddies, and if ${-1/3<\kappa _{\scriptscriptstyle {\varvec{\mathcal {S}}}^{\mathcal {A}},\varvec{\Omega }^{{{\mathcal {A}}}}}<1/3}$, the region is dominated by shear. As shown in Fig. 5a, the flow becomes increasingly shear-dominated near the carrier-liquid interface. This is quantified by plotting the histograms of the flow topology parameter ${\kappa _{\scriptscriptstyle {\varvec{\mathcal {S}}}^{\mathcal {A}},\varvec{\Omega }^{{{\mathcal {A}}}}}}$ for different regions, as shown in Fig. 5b. All calculated curves exhibit a dominant peak in correspondence at ${\kappa _{\scriptscriptstyle {\varvec{\mathcal {S}}}^{\mathcal {A}},\varvec{\Omega }^{{{\mathcal {A}}}}}=0}$, suggesting a dominantly shear flow. Close to the carrier-liquid interface, the right tail drops to zero for ${\kappa _{\scriptscriptstyle {\varvec{\mathcal {S}}}^{\mathcal {A}},\varvec{\Omega }^{{{\mathcal {A}}}}}=1}$ more quickly than for the other two regions, on the other hand, when far from the interface, the PDFs of ${\kappa _{\scriptscriptstyle {\varvec{\mathcal {S}}}^{\mathcal {A}},\varvec{\Omega }^{{{\mathcal {A}}}}}}$ indicate the increased presence of purely elongational flow.

### ${(Q^{{{\mathcal {A}}}},R^{{{\mathcal {A}}}})}$ diagrams

**Figure 6:**
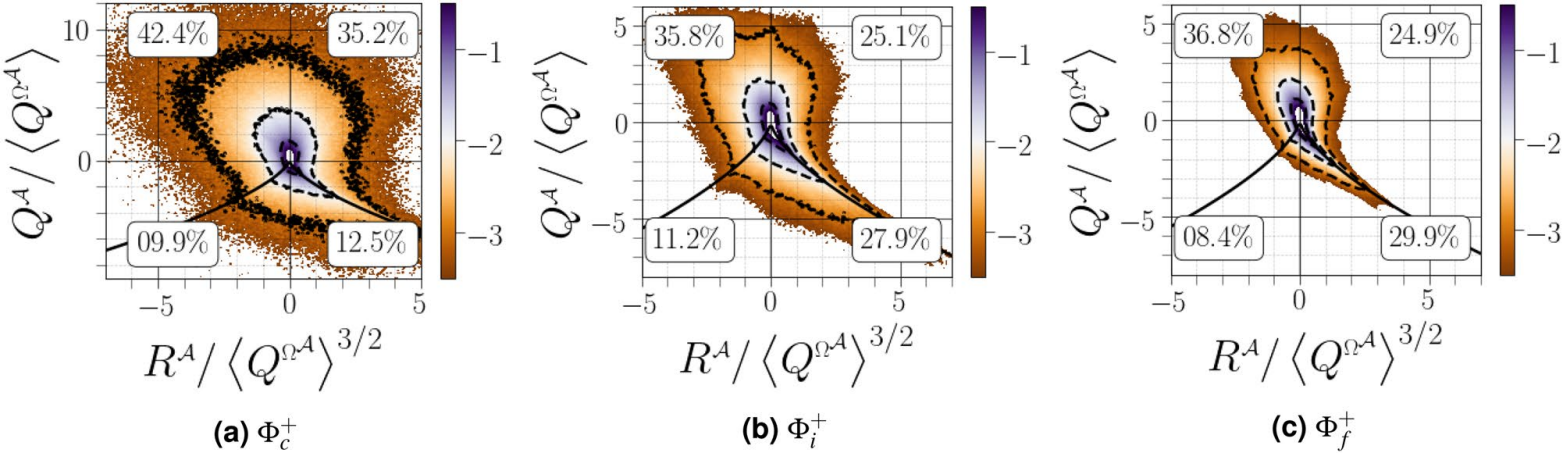
Joint $\text {PDF}$s of ${R^{{{\mathcal {A}}}}}$ *versus* ${Q^{{{\mathcal {A}}}}}$ conditioned on different distances from the interface. The three thin black lines correspond to isocontours equals to $-3$, $-2$, and $-1$. The bold numbers correspond to the ratios of the characteristic flow topologies in each of the four quadrants.

The joint PDFs of the ${(R^{{{\mathcal {A}}}},Q^{{{\mathcal {A}}}})}$ map exhibit a characteristic teardrop shape at ${\Phi }^{+}_f$ with narrow cusp in ${R^{{{\mathcal {A}}}}=0}$ and ${\Delta ^{{{\varvec{\mathcal {A}}}}}=0}$ (see Fig. 6c), in which ${Q^{{{\mathcal {A}}}}}$ and ${R^{{{\mathcal {A}}}}}$ are particularity well-correlated in the following regions: i) the upper left quadrant SFS (${Q^{{{\mathcal {A}}}}>0}$ and ${R^{{{\mathcal {A}}}}<0}$) indicating a predominance of biaxial stretching of the fluid elements and ii) the lower right quadrant UNSS (${Q^{{{\mathcal {A}}}}<0}$ and ${R^{{{\mathcal {A}}}}>0}$) associated with a predominance of enstrophy production by vortex stretching. The observed teardrop shape has previously been observed in single phase HIT, boundary layers, mixing layers and channel flows^28^. The modification of turbulence outside the liquid boundary layer is undetected when looking only at fine scale-scale motions. The isocontours lines corresponding to a constant probability density tend to broaden and increase monotonically as the carrier-liquid interface is approached (see Fig. 6a and b), indicating an increase in magnitude of velocity gradient due to the liquid–gas interface inertia causing deviations in gaseous velocity and, thus, increased gaseous velocity gradient. Note also that i) an increase in the correlation between ${Q^{{{\mathcal {A}}}}}$ and ${R^{{{\mathcal {A}}}}}$ in the UFC and SNSS was observed when approaching the liquid–gas interface and ii) there is an almost equal probability of enstrophy-dominated regions (UFC and SFS), leading to symmetric $(R^{{\mathcal {A}}},Q^{{\mathcal {A}}})$ map, similar to the single phase high-expansion regions noted in forced compressible HIT^28^ and shocked turbulence^29^. Recently, Hasslberger et al.^10^ proposed a model predicting that the total percentage of volume fraction is expected to be roughly 80% for focal topologies (40% for UFC and 40% for SFS) and 20% for nodal topologies (10% for SNSS and 10% for UNSS). The configuration investigated in the present study deviates slightly from the idealized model but can be considered approximatively valid for comparison purposes. We also noticed that, in terms of the probability of occurrence in each station, the region ${\Phi }_i^{+}$ exhibits a transitional shape with a more pronounced tail than at ${\Phi }^{+}$ and larger velocity gradients than at ${\Phi }_f^{+}$. Similar symmetric behaviour between ${R^{{{\mathcal {A}}}}}$ and ${Q^{{{\mathcal {A}}}}}$ was reported by Hasslberger et al.^11^ for bubble induced turbulence. Moreover, the analytical solution around a bubble obeying Stokes flow yields qualitatively similar behaviour.

### ${(Q^{{{\mathcal {S}}}^{{{\mathcal {A}}}}},R^{{{\mathcal {S}}}^{{{\mathcal {A}}}}})}$ diagrams

**Figure 7:**
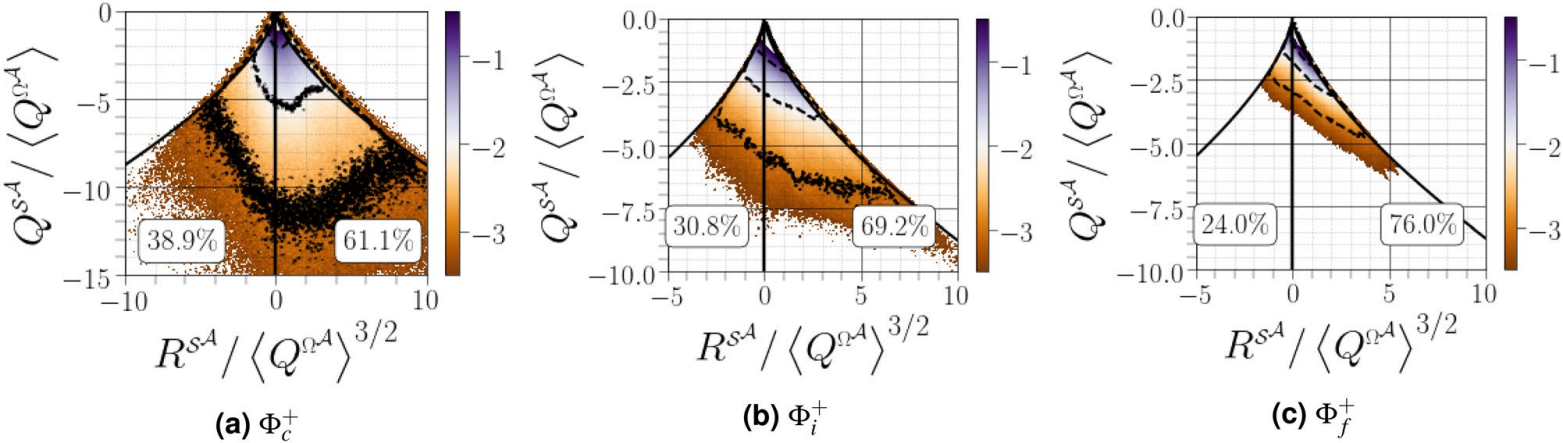
Joint $\text {PDF}$s of ${R^{{{\mathcal {S}}}^{{{\mathcal {A}}}}}}$ *versus* ${Q^{{{\mathcal {S}}}^{{{\mathcal {A}}}}}}$ conditioned on different distances from the interface. The three thin black lines correspond to isocontours equals to $-3$, $-2$, and $-1$. The bold numbers correspond to ${R^{{{\mathcal {S}}}^{{{\mathcal {A}}}}}>0}$ (right value) and ${R^{{{\mathcal {S}}}^{{{\mathcal {A}}}}}<0}$ (left value).

The joint PDFs of ${(Q^{{{\mathcal {S}}}^{{{\mathcal {A}}}}},R^{{{\mathcal {S}}}^{{{\mathcal {A}}}}})}$ are depicted in Fig. 7 in order to analyse the geometry of straining of the fluid element in every station. Equation (2) indicates that locations with ${Q^{{{\mathcal {S}}}^{{{\mathcal {A}}}}}\le 0}$ are associated with high level of dissipation. Moreover, equation (3) implies that ${R^{{{\mathcal {S}}}^{{{\mathcal {A}}}}}}$ has the same sign as $\lambda _2$. Thus, ${R^{{{\mathcal {S}}}^{{{\mathcal {A}}}}}>0}$ (resp. ${R^{{{\mathcal {A}}}}<0}$) implies the production (resp. destruction) of ${\mathcal {\varvec{S}}^{{{{\mathcal {A}}},2}}}$, and is thus associated with sheetlike structure (resp. tubelike structures). The most striking feature of the joint PDFs of ${(Q^{{{\mathcal {S}}}^{{{\mathcal {A}}}}},R^{{{\mathcal {S}}}^{{{\mathcal {A}}}}})}$ is the decreasing preference for a rate-of-strain topology of the type saddle-saddle-unstable-node (two positive eigenvalues and one negative, *i.e.*, expansion of fluid elements) when approaching the liquid–gas interface (see Fig. 7b, c). The probability of finding ${R^{{{\mathcal {S}}}^{{{\mathcal {A}}}}}>0}$ becomes slightly greater than finding ${R^{{{\mathcal {S}}}^{{{\mathcal {A}}}}}<0}$ in the vicinity of the carrier-liquid interface and, thus, the joint PDF of ${(R^{{{\mathcal {S}}}^{{{\mathcal {A}}}}},Q^{{{\mathcal {S}}}^{{{\mathcal {A}}}}})}$ becomes slightly tilted towards ${R^{{{\mathcal {S}}}^{{{\mathcal {A}}}}}>0}$ and tends to be symmetric along ${R^{{{\mathcal {S}}}^{{{\mathcal {A}}}}}=0}$ as seen in Fig. 7a; this implies the presence of more contraction in the region. It is also notable that the lines of constant probability increases when approaching the carrier-liquid interface, resulting in a higher flow dissipation rate close to the interface. Mapping of the joint PDFs of ${Q^{{{\mathcal {S}}}^{{{\mathcal {A}}}}}}$ and ${R^{{{\mathcal {S}}}^{{{\mathcal {A}}}}}}$ attains, at least qualitatively, the characteristic shape, for example, in single-phase mixing layers^30^, channel flow^15^, and homogeneous isotropic turbulence^31^. This diagram highlights that the information gleaned from the ${(Q^{{{\mathcal {A}}}},R^{{{\mathcal {A}}}})}$ diagram is insufficient to characterize turbulent flows since the similar *’teardrop structures’* masks important turbulence information.

### ${(Q^{{\Omega }^{{{\mathcal {A}}}}},-Q^{{{\mathcal {S}}}^{{{\mathcal {A}}}}})}$ diagrams

**Figure 8:**
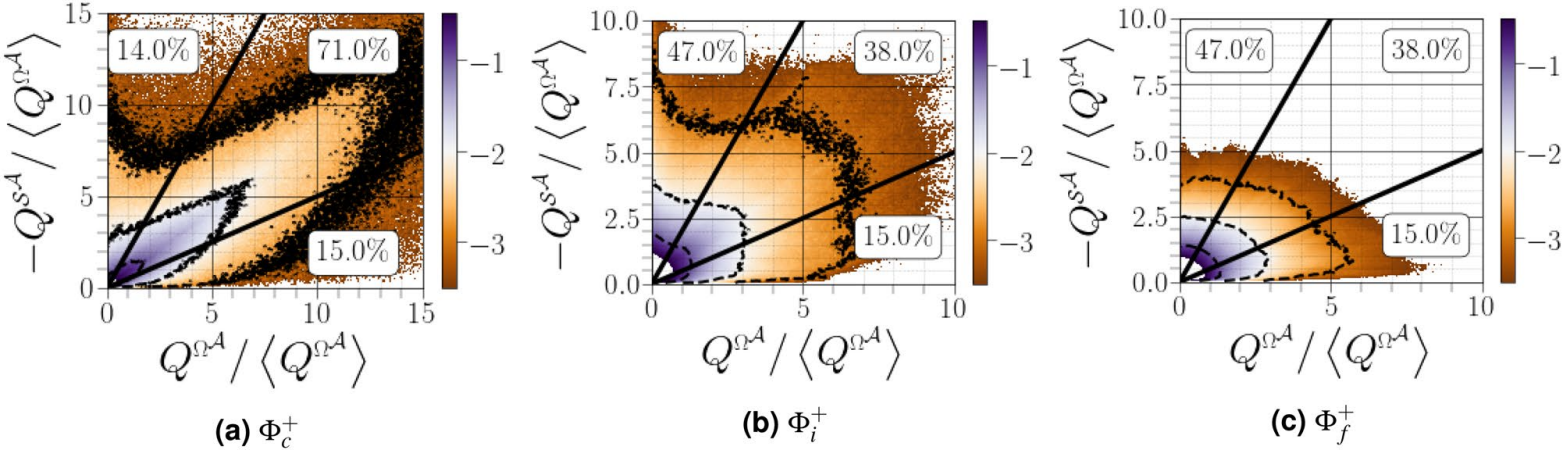
Joint $\text {PDF}$s of ${Q^{{\Omega }^{{{\mathcal {A}}}}}}$ *versus* -${Q^{{{\mathcal {S}}}^{{{\mathcal {A}}}}}}$ conditioned on different distances from the interface. The three thin black lines correspond to iso-contours equals to $-3$, $-2$, and $-1$. The convergence, shear and eddy regions are separated by dashed lines. Bold numbers correspond to the percentage of each region.

The diagram of ${(Q^{{\Omega }^{{{\mathcal {A}}}}},-Q^{{{\mathcal {S}}}^{{{\mathcal {A}}}}})}$ maps shown in Fig. 8 indicate that the bulk of the data tend to be concentrated near the origin, whereas high gradient motions are distributed differently, *i.e.*, as functions of distance from the carrier-liquid interface. In particular, *in the vicinity of* the interface, most data fall on the $45^{\circ }$ line through the origin, representing a region in the flow with high dissipation accompanied by high enstrophy density (see Fig. 8a). This is consistent with Fig. 7a, which shows a local vortex sheet, *i.e.*, the rate of strain is dominated by the velocity gradient within the sheet. In the closest region from the carrier-liquid interface, the joint PDF of ${(Q^{{\Omega }^{{{\mathcal {A}}}}},-Q^{{{\mathcal {S}}}^{{{\mathcal {A}}}}})}$ shows a marked tendency to be aligned with the vertical line defined by ${Q^{{\Omega }^{{{\mathcal {A}}}}}=0}$. This attests to a predominance of dissipation (strain production) over enstrophy, *i.e.*, irrotational dissipation. Far from the liquid-gas interface, the peak value of ${Q^{{\Omega }^{{{\mathcal {A}}}}}}$ reaches larger values than the peak value of ${Q^{{{\mathcal {S}}}^{{{\mathcal {A}}}}}}$ within the same probability contour (this was also observed in single phase HIT^31^), whereas the opposite occurs close to the interface (a similar behavior was observed in the log-law and wake regions of channel flow simulations by Blackburn et al.^15^).

To more accurately analyse the contribution each topology of ${(Q^{{\Omega }^{{{\mathcal {A}}}}},-Q^{{{\mathcal {S}}}^{{{\mathcal {A}}}}})}$ maps on the gaseous flowfield, Kevlahan et al.^32^ introduced three different flow regions based on enstrophy and dissipation: (i) the convergence region, ${Q^{{\Omega }^{{{\mathcal {A}}}}}<-Q^{{{\mathcal {S}}}^{{{\mathcal {A}}}}}/2}$, where irrotational strain is high compared with the rotational strain, (ii) the shear region, ${-Q^{{{\mathcal {S}}}^{{{\mathcal {A}}}}}/2\le Q^{{\Omega }^{{{\mathcal {A}}}}}\le -2Q^{{{\mathcal {S}}}^{{{\mathcal {A}}}}}}$, where irrotational and rotational strain are approximately equal, and (iii) eddy region, ${Q^{{\Omega }^{{{\mathcal {A}}}}}>-2Q^{{{\mathcal {S}}}^{{{\mathcal {A}}}}}}$, where rotational straining is high compared with irrotational straining. Far from the carrier-liquid interface, 20% of the flow volume is in the eddy zone, 45% in the shear zone and 35% in the convergence zone. These values are quite similar to those in the joint PDFs obtained for single-phase incompressible HIT by Ooi et al.^31^ (the percentage of eddy, shear and convergence zones in that study were 19.2%, 44.6% and 36.2%, respectively). *In the vicinity of the interface, both eddy and convergence regions have equal probabilities, while most data fall within the shear region.*

### Normal and non-normal effects on the velocity gradient dynamics

**Figure 9:**
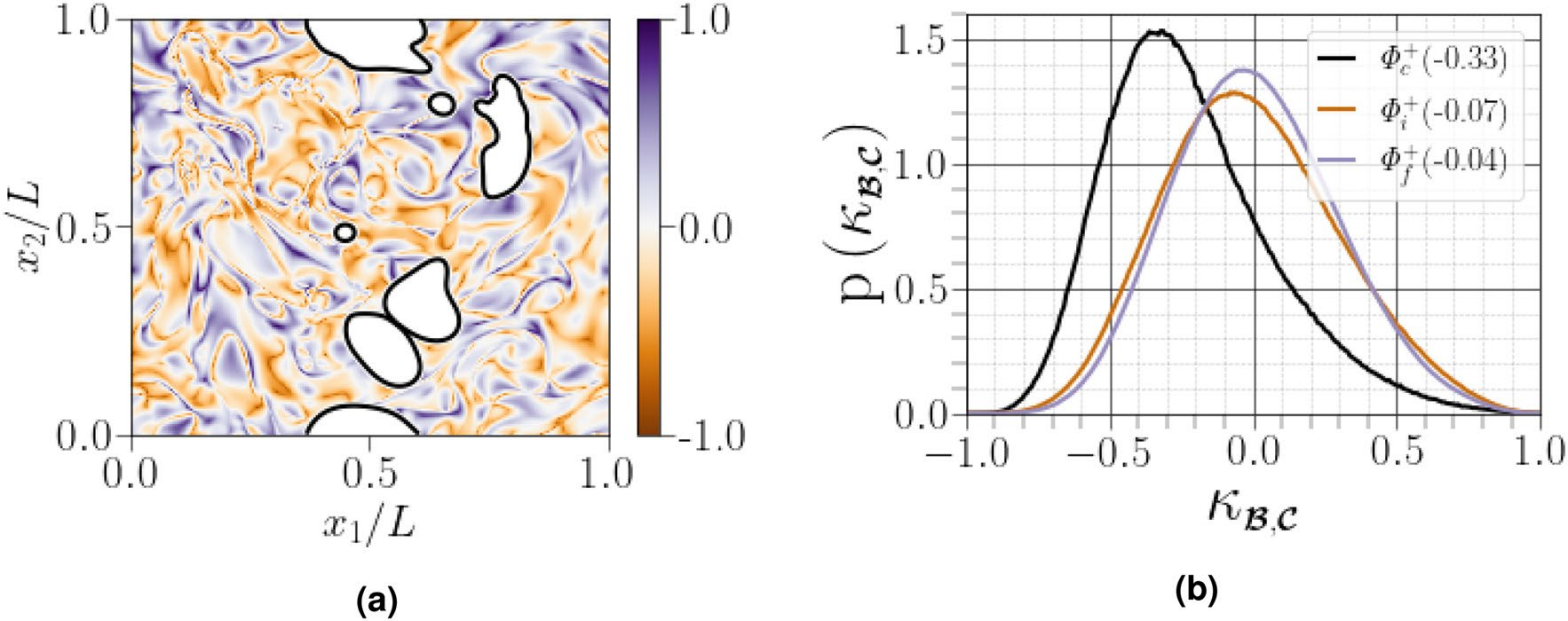
(**a**) Distribution of ${\kappa _{{\varvec{\mathcal {B}}},{\varvec{\mathcal {C}}}}}$ in $x_3=L/2$ slice, black spots corresponds to the liquid phase, (**b**) PDF curves for ${\kappa _{{\varvec{\mathcal {B}}},{\varvec{\mathcal {C}}}}}$ conditioned on different distances from the interface. Figures in parentheses indicate the mode of the corresponding PDF curve.

We first estimated the relative importance of the normal and non-normal contributions for the decomposition given by ${{\varvec{\mathcal {A}}}={\varvec{\mathcal {B}}}+{\varvec{\mathcal {C}}}}$. For ${\Vert {\varvec{\mathcal {C}}}\Vert \approx 0}$, it is legitimate to model the behaviour of ${{\varvec{\mathcal {A}}}}$ using its eigenvalues. For the flow topology parameter ${\kappa _{\scriptscriptstyle {\varvec{\mathcal {S}}}^{\mathcal {A}},\varvec{\Omega }^{{{\mathcal {A}}}}}}$, we plot in Fig. 9 the standardized difference ${\kappa _{{\varvec{\mathcal {B}}},{\varvec{\mathcal {C}}}}=(\Vert {\varvec{\mathcal {B}}}\Vert -\Vert {\varvec{\mathcal {C}}}\Vert )/(\Vert {\varvec{\mathcal {B}}}\Vert +\Vert {\varvec{\mathcal {C}}}\Vert )}$ to estimate the contributions of normal and non-normal effects to the dynamic of the velocity gradient. Normal effects are considered to be important when values of ${\kappa _{{\varvec{\mathcal {B}}},{\varvec{\mathcal {C}}}}}$ are close to 1, whereas they become negligible as ${\kappa _{{\varvec{\mathcal {B}}},{\varvec{\mathcal {C}}}}}$ approaches $-1$. In the gaseous flow field far from the carrier-liquid interface, the overall mode of the distribution of ${\kappa _{{\varvec{\mathcal {B}}},{\varvec{\mathcal {C}}}}}$ was slightly negative, with a median close to zero, indicating that the non-normal effects in the dynamics of two-phase homogeneous isotropic turbulent flow are significant to ${{\varvec{\mathcal {A}}}}$ relative to the eigenvalues. Similar tendencies have previously been noted for single phase HIT^13^. However, there is a tendency for more strongly negative values of ${\kappa _{{\varvec{\mathcal {B}}},{\varvec{\mathcal {C}}}}}$ close to the carrier-liquid interface, suggesting that tensor non-normality plays a greater role in these dynamics when moving into the boundary layer close to the carrier-liquid interface.

**Figure 10:**
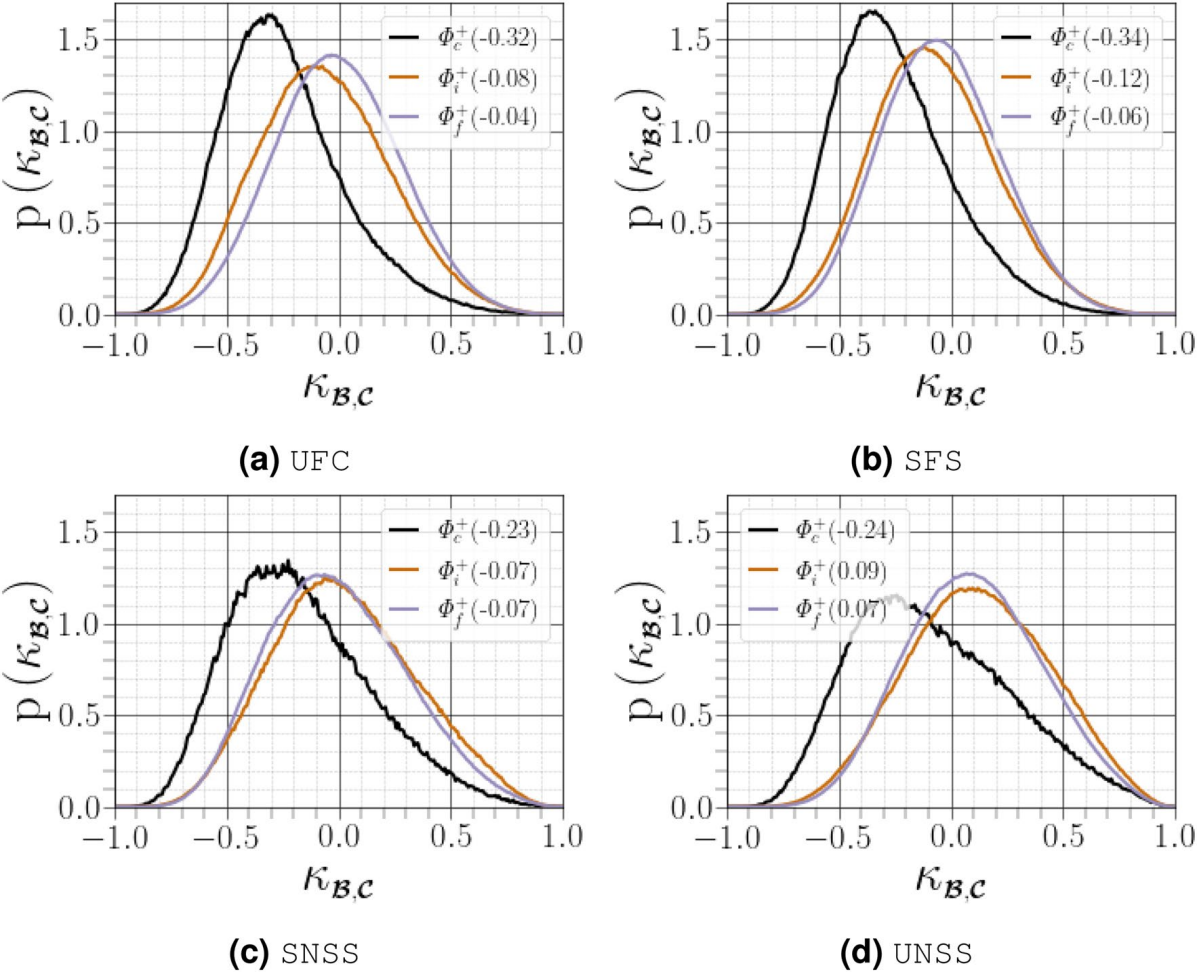
PDFs for the normality/non-normality index ${\kappa _{{\varvec{\mathcal {B}}},{\varvec{\mathcal {C}}}}}$ as a function of their region in ${(R^{{{\mathcal {A}}}},Q^{{{\mathcal {A}}}})}$ diagram. Figures in parentheses indicate the mode of the corresponding PDF curve.

**Figure 11:**
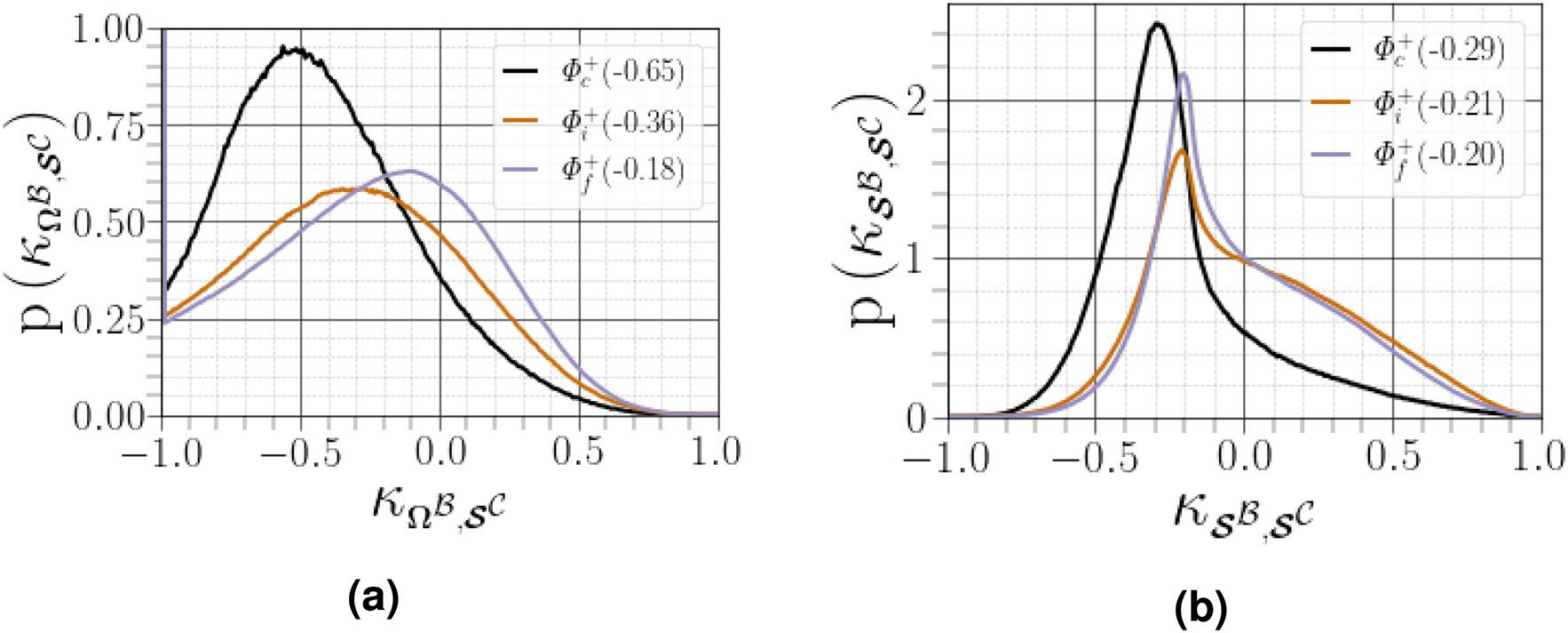
PDFs for (**a**) ${\kappa _{\scriptscriptstyle \varvec{\Omega }^{{\mathcal {B}}},\scriptscriptstyle {\varvec{\mathcal {S}}}^{{\mathcal {C}}}}}$ and (**b**) ${\kappa _{\scriptscriptstyle {\varvec{\mathcal {S}}}^{{\mathcal {B}}},\scriptscriptstyle {\varvec{\mathcal {S}}}^{{\mathcal {C}}}}}$ conditioned on different distances from the interface. Figures in parentheses indicate the mode of the corresponding PDF curve, where data for ${\kappa _{\scriptscriptstyle \varvec{\Omega }^{{\mathcal {B}}},\scriptscriptstyle {\varvec{\mathcal {S}}}^{{\mathcal {C}}}}=-1}$ are excluded.

When conditioning the overall PDF of ${\kappa _{{\varvec{\mathcal {B}}},{\varvec{\mathcal {C}}}}}$ by four regions of joint PDF of ${(R^{{{\mathcal {A}}}},Q^{{{\mathcal {A}}}})}$, as shown in Fig. 10, we observed that i) the contribution of ${\Vert {\varvec{\mathcal {C}}}\Vert }$ becomes higher where ${R^{{{\mathcal {A}}}}<0}$ regardless of the distance from the interface, ii) the departure from the zero mode was reduced far from the interface, and iii) the left skewness of the PDF was less pronounced in the region UNSS far from the interface, denoting that a purely eigenvalue-based description of the flow field is effective near the Vieillefosse tail (${R^{{{\mathcal {A}}}}>0}$ and ${\Delta ^{{{\varvec{\mathcal {A}}}}}=0}$). This observation is consistent with the findings of Cantwell^33^ and Keylock^13^. Non-normal effects dominate close to the carrier-liquid interface in all four region of the ${(R^{{{\mathcal {A}}}},Q^{{{\mathcal {A}}}})}$ map, in particular for the SFS and UFC regions where they present the most strongly negative mode. On the other hand, the UNSS region presents the most positive mode. The latter result is the most striking since ${\Delta ^{{{\varvec{\mathcal {A}}}}}<0}$ and ${\Vert \varvec{\Omega }^{{{\mathcal {B}}}}\Vert =0}$, probably indicating that ${\Vert {\varvec{\mathcal {S}}}^{{{\mathcal {B}}}}\Vert >\Vert {\varvec{\mathcal {S}}}^{{{\mathcal {C}}}}\Vert +\Vert \varvec{\Omega }^{{{\mathcal {C}}}}\Vert }$, or ${\Vert {\varvec{\mathcal {S}}}^{{{\mathcal {B}}}}\Vert >2\Vert {\varvec{\mathcal {S}}}^{{{\mathcal {C}}}}\Vert }$. Therefore, adopting an eigenvalue-based description of the flow is essentially effective in the vicinity of the Vieillefosse tail in the lower hand quadrant of the ${(Q^{{{\mathcal {A}}}},R^{{{\mathcal {A}}}})}$ plane, which acts as an attractor for the dynamics of the restricted Euler (eigenvalue-based) dynamics of ${{\varvec{\mathcal {A}}}}$ (which amounts to neglecting the anisotropic portion of the pressure Hessian tensor)^13,33^. To evaluate the effect of non-normality on the production of enstrophy and dissipation presented in (12), we again used the standardized differences ${\kappa _{\scriptscriptstyle \varvec{\Omega }^{{\mathcal {B}}},\scriptscriptstyle {\varvec{\mathcal {S}}}^{{\mathcal {C}}}}=(\Vert \varvec{\Omega }^{{{\mathcal {B}}}}\Vert -\Vert {\varvec{\mathcal {S}}}^{{{\mathcal {C}}}}\Vert )/(\Vert \varvec{\Omega }^{{{\mathcal {B}}}}\Vert +\Vert {\varvec{\mathcal {S}}}^{{{\mathcal {C}}}}\Vert )}$ and ${\kappa _{\scriptscriptstyle {\varvec{\mathcal {S}}}^{{\mathcal {B}}},\scriptscriptstyle {\varvec{\mathcal {S}}}^{{\mathcal {C}}}}=(\Vert {\varvec{\mathcal {S}}}^{{{\mathcal {B}}}}\Vert -\Vert {\varvec{\mathcal {S}}}^{{{\mathcal {C}}}}\Vert )/(\Vert {\varvec{\mathcal {S}}}^{{{\mathcal {B}}}}\Vert +\Vert {\varvec{\mathcal {S}}}^{{{\mathcal {C}}}}\Vert )}$ (see Fig. 11). Note that ${\Vert \varvec{\Omega }^{{{\mathcal {B}}}}\Vert =0}$ throughout the SNSS and UNSS regions (${\Delta ^{{{\varvec{\mathcal {A}}}}}<0}$), and ${\Vert \varvec{\Omega }^{{{\mathcal {C}}}}\Vert =\Vert {\varvec{\mathcal {S}}}^{{{\mathcal {B}}}}\Vert }$. The dynamics of the rate-of-rotation and rate-of-strain tensors are dictated only by the non-normal part, ${\Vert {\varvec{\mathcal {C}}}\Vert }$, particularly when close to the carrier-liquid interface, as inferred by the strong negative skewness of both ${\kappa _{\scriptscriptstyle {\varvec{\mathcal {S}}}^{{\mathcal {B}}},\scriptscriptstyle {\varvec{\mathcal {S}}}^{{\mathcal {C}}}}}$ and ${\kappa _{\scriptscriptstyle \varvec{\Omega }^{{\mathcal {B}}},\scriptscriptstyle {\varvec{\mathcal {S}}}^{{\mathcal {C}}}}}$. This suggests that the properties of vortical structures reported in the literature, such as topology and alignment, are poorly described by the eigenvalues and depend on non-normal contributions.

**Figure 12:**
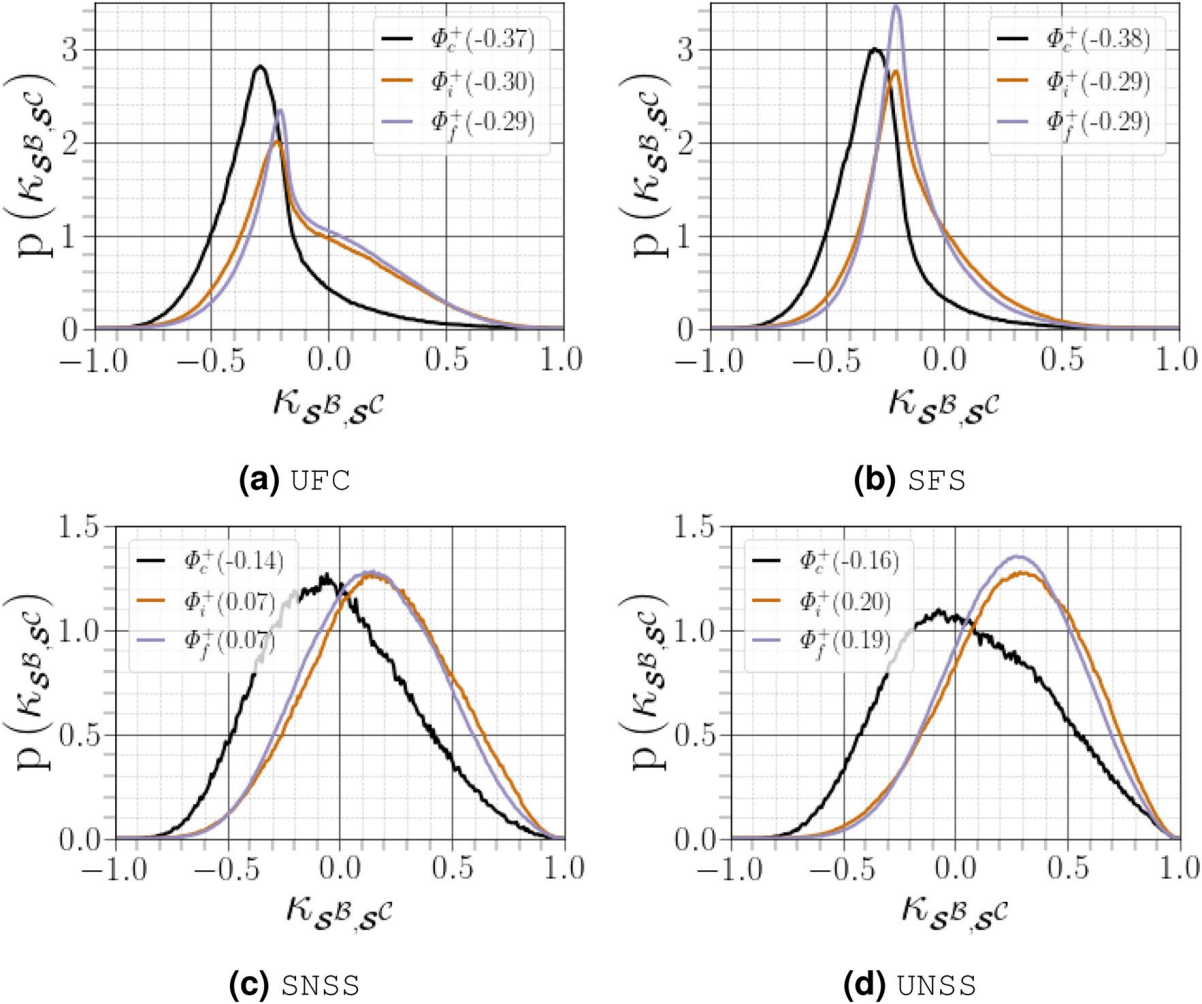
PDFs of ${\kappa _{\scriptscriptstyle {\varvec{\mathcal {S}}}^{{\mathcal {B}}},\scriptscriptstyle {\varvec{\mathcal {S}}}^{{\mathcal {C}}}}}$ as a function of region of the ${(R^{{{\mathcal {A}}}},Q^{{{\mathcal {A}}}})}$ diagram. Figures in parentheses indicate the mode of the corresponding PDF curve.

When the results of ${\kappa _{\scriptscriptstyle {\varvec{\mathcal {S}}}^{{\mathcal {B}}},\scriptscriptstyle {\varvec{\mathcal {S}}}^{{\mathcal {C}}}}}$ are partitioned by the four regions of ${(R^{{{\mathcal {A}}}},Q^{{{\mathcal {A}}}})}$ space (see Fig. 12), we found that when ${R^{{{\mathcal {A}}}}<0}$, the distribution of ${{\varvec{\mathcal {S}}}^{{{\mathcal {C}}}}}$ dominates over that of ${R^{{{\mathcal {A}}}}>0}$ (consistent with the modes of ${\kappa _{\scriptscriptstyle {\varvec{\mathcal {S}}}^{{\mathcal {B}}},\scriptscriptstyle {\varvec{\mathcal {S}}}^{{\mathcal {C}}}}}$). As anticipated from the analysis of Fig. 11b, we observed a difference between the SNSS and UNSS regions in terms of their modal values of ${\kappa _{\scriptscriptstyle {\varvec{\mathcal {S}}}^{{\mathcal {B}}},\scriptscriptstyle {\varvec{\mathcal {S}}}^{{\mathcal {C}}}}}$, which were 0.07 and 0.19, respectively. Close to the carrier-liquid interface, the non-normal effects are predominant throughout the entirety of ${(R^{{{\mathcal {A}}}},Q^{{{\mathcal {A}}}})}$ space, with the least prominent effects found in regions characterized by ${\Delta ^{{{\varvec{\mathcal {A}}}}}<0}$. Overall, when enstrophy production dominates, there is a greater probability for larger shear effects than the eigenvalue-associated effects related to ${\varvec{\mathcal {S}}}^{\mathcal {A}}$ and $\varvec{\Omega }^{{{\mathcal {A}}}}$.

### Factors affecting enstrophy production and strain self-amplification

In this section, we analyse the factors that influence important velocity gradient terms, in particular, enstrophy production and strain self amplification. For incompressible flows, the alignment between vorticity ${\varvec{\omega }^{{{\mathcal {A}}}}}$ and the eigenvectors of the strain-rate tensor ${\varvec{\mathcal {S}}}^{\mathcal {A}}$ is presumably the primary mechanism controlling the transfer of turbulent kinetic energy from large scales to small ones through vortex stretching^34^. Analyses of the ${\varvec{\omega }^{{{\mathcal {A}}}}-{\varvec{\mathcal {S}}}^{\mathcal {A}}}$ coupling are therefore required to understand the nature of fluctuations in strain-rate in turbulent flow. The evolution of vorticity and strain fields are strongly connected in a non-linear manner. In particular, the dynamics of both fields are primarily driven by self-amplification demonstrated by the transport equations for enstrophy and strain-rate norm squared as follows^35^:

18 $$\begin{aligned} {\frac{1}{2}\frac{D\left( \omega ^{{\mathcal {A}}}_i\omega ^{{\mathcal {A}}}_i\right) }{D t} = \omega ^{{\mathcal {A}}}_i\omega ^{{\mathcal {A}}}_j{\mathcal {S}}^{{{\mathcal {A}}}}_{ij}-\omega ^{{\mathcal {A}}}_i\omega ^{{\mathcal {A}}}_j{\mathcal {S}}_{kk}^{{\mathcal {A}}}\delta _{ij}+ \frac{\omega ^{{\mathcal {A}}}_i \varepsilon   _{ijk}}{\rho ^2} \frac{\partial \rho }{\partial x_j} \frac{\partial {\mathfrak {p}}}{\partial x_k} +\omega ^{{\mathcal {A}}}_i \varepsilon   _{ijk} \frac{\partial }{\partial x_j} \left( \frac{1}{\rho } \frac{\partial \tau _{kl}}{\partial x_l} \right) +{\mathcal {T}}_{\text {cap}},} \end{aligned}$$

where ${\tau _{ij}=2\mu {\mathcal {S}}^{{{\mathcal {A}}}}_{ij}}$ is the viscous stress tensor and ${\mathcal {T}}_{\text {cap}}$ is an extra potential enstrophy production term, which occurs due to the the capillary force itself^11^, and

19 $$\begin{aligned} {\frac{1}{2}\frac{D\left( {\mathcal {S}}^{{{\mathcal {A}}}}_{ii}{\mathcal {S}}^{{{\mathcal {A}}}}_{ii}\right) }{D t}=-{\mathcal {S}}^{{{\mathcal {A}}}}_{ij}{\mathcal {S}}^{{{\mathcal {A}}}}_{jk}{\mathcal {S}}^{{{\mathcal {A}}}}_{ki}-\frac{1}{4}\omega ^{{\mathcal {A}}}_i\omega ^{{\mathcal {A}}}_j{\mathcal {S}}^{{{\mathcal {A}}}}_{ij}-{\mathcal {S}}^{{{\mathcal {A}}}}_{ij}\frac{\partial ^2{\mathfrak {p}}}{\partial x_i\partial x_j}+\nu {\mathcal {S}}^{{{\mathcal {A}}}}_{ij}\varvec{\nabla }^2{\mathcal {S}}^{{{\mathcal {A}}}}_{ij}.} \end{aligned}$$

Note that equations (18) and (19) are valid only for single phase flows without interfaces. The production of enstrophy and strain are both dominated by the terms ${\omega ^{{{\mathcal {A}}}}_i\omega ^{{{\mathcal {A}}}}_j{\mathcal {S}}^{{{\mathcal {A}}}}_{ij}}$ and ${{\mathcal {S}}^{{{\mathcal {A}}}}_{ij}{\mathcal {S}}^{{{\mathcal {A}}}}_{jk}{\mathcal {S}}^{{{\mathcal {A}}}}_{ki}}$, which are expressed using the eigenvectors and eigenvalues of ${\varvec{\mathcal {S}}}^{\mathcal {A}}$ as follows:

20 $$\begin{aligned} {\omega ^{{{\mathcal {A}}}}_i\omega ^{{{\mathcal {A}}}}_j{\mathcal {S}}^{{{\mathcal {A}}}}_{ij}=\Vert \varvec{\omega }^{{{\mathcal {A}}}}\Vert ^2\Vert {\varvec{\mathcal {S}}}^{\mathcal {A}}\Vert \mathring{\lambda ^{{{\mathcal {A}}}}_i}\cos ^2\left( \mathbf{e }^{{\mathcal {A}}}_i,\mathring{\varvec{\omega }^{{{\mathcal {A}}}}}\right) } \end{aligned}$$

and

21 $$\begin{aligned} {{\mathcal {S}}^{{{\mathcal {A}}}}_{ij}{\mathcal {S}}^{{{\mathcal {A}}}}_{jk}{\mathcal {S}}^{{{\mathcal {A}}}}_{ki}=\Vert {\varvec{\mathcal {S}}}^{\mathcal {A}}\Vert ^3\left( \mathring{\lambda ^{{{\mathcal {A}}}}_i}\right) ^3,} \end{aligned}$$

where ${\mathring{\varvec{\omega }^{{{\mathcal {A}}}}}=\varvec{\omega }^{{{\mathcal {A}}}}/\Vert \varvec{\omega }^{{{\mathcal {A}}}}\Vert }$ is the normalized vorticity vector, ${|\cos \left( \mathbf{e }^{{\mathcal {A}}}_i,\mathring{\varvec{\omega }^{{{\mathcal {A}}}}}\right) |}$, denoted hereafter as $\zeta ^{\mathbf{e }^{{\mathcal {A}}}_i,\varvec{\omega }^{{\mathcal {A}}}}$, corresponding to the alignment between the vorticity vector for ${\varvec{\omega }^{{{\mathcal {A}}}}}$ and the $i^\text {th}$ eigenvector for the strain rate tensor of ${{\varvec{\mathcal {A}}}}$, such that ${\mathring{\lambda ^{{{\mathcal {A}}}}_i}}$ and $\mathbf{e }^{{\mathcal {A}}}_i$ are the normalized eigenvalues and eigenvectors of ${\varvec{\mathcal {S}}}^{\mathcal {A}}$, respectively. The eigenvectors are scaled to unit length, whereas the eigenvalues are normalized by the magnitude of strain rate ${\mathring{\lambda ^{{{\mathcal {A}}}}_i}=\lambda ^{{{\mathcal {A}}}}_i/\Vert {\varvec{\mathcal {S}}}^{\mathcal {A}}\Vert }$. The eigenvalues are purely real (because ${\varvec{\mathcal {S}}}^{\mathcal {A}}$ is symmetric) and are in decreasing order: ${\lambda ^{{{\mathcal {A}}}}_1\ge \lambda ^{{{\mathcal {A}}}}_2\ge \lambda ^{{{\mathcal {A}}}}_3}$. Furthermore, the relation ${\varvec{\nabla }\cdot {\mathfrak {u}}=\lambda ^{{{\mathcal {A}}}}_i}$ holds, in the case of incompressible flows this leads to ${\lambda ^{{{\mathcal {A}}}}_1>0}$ and ${\lambda ^{{{\mathcal {A}}}}_3<0}$. Incompressibility also dictates that the maximum value obtained by any one normalised strain rate is ${\mathring{\lambda ^{{{\mathcal {A}}}}_i}=\pm 2/\sqrt{6}}$.

**Figure 13:**
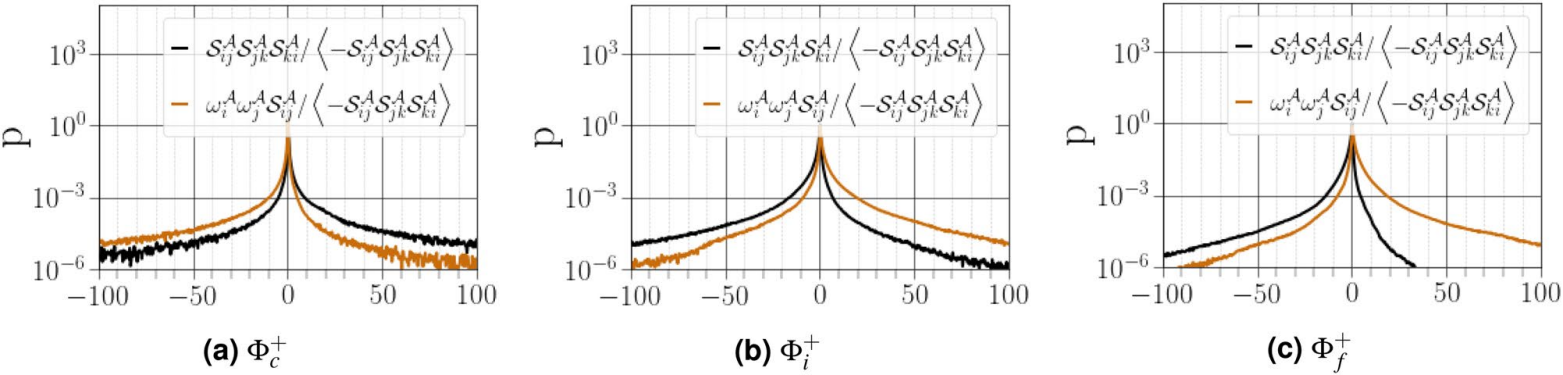
PDFs of ${\omega ^{{{\mathcal {A}}}}_i\omega ^{{{\mathcal {A}}}}_j{\mathcal {S}}^{{{\mathcal {A}}}}_{ij}}$ and ${{\mathcal {S}}^{{{\mathcal {A}}}}_{ij}{\mathcal {S}}^{{{\mathcal {A}}}}_{jk}{\mathcal {S}}^{{{\mathcal {A}}}}_{ki}}$ normalized by ${\left\langle -{\mathcal {S}}^{{{\mathcal {A}}}}_{ij}{\mathcal {S}}^{{{\mathcal {A}}}}_{jk}{\mathcal {S}}^{{{\mathcal {A}}}}_{ki}\right\rangle }$ conditioned on different distances from the interface.

The PDFs of enstrophy production ${\omega ^{{{\mathcal {A}}}}_i\omega ^{{{\mathcal {A}}}}_j{\mathcal {S}}^{{{\mathcal {A}}}}_{ij}}$ and strain self-amplification ${{\mathcal {S}}^{{{\mathcal {A}}}}_{ij}{\mathcal {S}}^{{{\mathcal {A}}}}_{jk}{\mathcal {S}}^{{{\mathcal {A}}}}_{ki}}$ both normalized by ${\left\langle -{\mathcal {S}}^{{{\mathcal {A}}}}_{ij}{\mathcal {S}}^{{{\mathcal {A}}}}_{jk}{\mathcal {S}}^{{{\mathcal {A}}}}_{ki}\right\rangle }$ (note that this term is always positive) are reported in Fig. 13. Close to the interface, the PDFs of both ${\omega ^{{{\mathcal {A}}}}_i\omega ^{{{\mathcal {A}}}}_j{\mathcal {S}}^{{{\mathcal {A}}}}_{ij}}$ and ${{\mathcal {S}}^{{{\mathcal {A}}}}_{ij}{\mathcal {S}}^{{{\mathcal {A}}}}_{jk}{\mathcal {S}}^{{{\mathcal {A}}}}_{ki}}$ are almost symmetric with negligible values. With increasing distance from the carrier-liquid interface, the PDF of ${{\mathcal {S}}^{{{\mathcal {A}}}}_{ij}{\mathcal {S}}^{{{\mathcal {A}}}}_{jk}{\mathcal {S}}^{{{\mathcal {A}}}}_{ki}}$ skews towards negative values, while the PDF of ${\omega ^{{{\mathcal {A}}}}_i\omega ^{{{\mathcal {A}}}}_j{\mathcal {S}}^{{{\mathcal {A}}}}_{ij}}$ skews towards positive values. The tendency observed at the interface signifies the prevalence of vortex stretching over vortex compression, which is considered to be a universal characteristic of small-scale features in turbulent flows^2^. A similar shape was noted in the inner layer of compressible turbulent boundary layers^36^.

**Figure 14:**
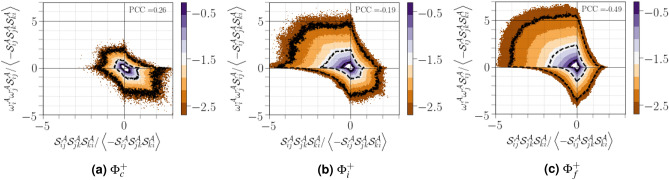
Joint PDFs of ${\omega ^{{{\mathcal {A}}}}_i\omega ^{{{\mathcal {A}}}}_j{\mathcal {S}}^{{{\mathcal {A}}}}_{ij}}$ and ${{\mathcal {S}}^{{{\mathcal {A}}}}_{ij}{\mathcal {S}}^{{{\mathcal {A}}}}_{jk}{\mathcal {S}}^{{{\mathcal {A}}}}_{ki}}$ normalized by ${\left\langle -{\mathcal {S}}^{{{\mathcal {A}}}}_{ij}{\mathcal {S}}^{{{\mathcal {A}}}}_{jk}{\mathcal {S}}^{{{\mathcal {A}}}}_{ki}\right\rangle }$ conditioned on different distances from the interface. The three thin black lines correspond to iso-contours equals to $-2.5$, $-1.5$, and $-0.5$ and the value at the top left corresponds to the Pearson correlation coefficient (PCC) between ${\omega ^{{{\mathcal {A}}}}_i\omega ^{{{\mathcal {A}}}}_j{\mathcal {S}}^{{{\mathcal {A}}}}_{ij}}$ and ${{\mathcal {S}}^{{{\mathcal {A}}}}_{ij}{\mathcal {S}}^{{{\mathcal {A}}}}_{jk}{\mathcal {S}}^{{{\mathcal {A}}}}_{ki}}$.

To understand the coupling between enstrophy production and strain self-amplification, we used the joint PDFs of ${\omega ^{{{\mathcal {A}}}}_i\omega ^{{{\mathcal {A}}}}_j{\mathcal {S}}^{{{\mathcal {A}}}}_{ij}}$ and ${{\mathcal {S}}^{{{\mathcal {A}}}}_{ij}{\mathcal {S}}^{{{\mathcal {A}}}}_{jk}{\mathcal {S}}^{{{\mathcal {A}}}}_{ki}}$ (see Fig. 14). Far from the interface, the contour lines suggests that the carrier gas have a propensity to be located in the top-left quadrant (*i.e.*, ${\omega ^{{{\mathcal {A}}}}_i\omega ^{{{\mathcal {A}}}}_j{\mathcal {S}}^{{{\mathcal {A}}}}_{ij}>0}$ and ${{\mathcal {S}}^{{{\mathcal {A}}}}_{ij}{\mathcal {S}}^{{{\mathcal {A}}}}_{jk}{\mathcal {S}}^{{{\mathcal {A}}}}_{ki}<0}$), demonstrating that strong production of enstrophy inhibits strong production of strain and vice versa. Moreover, for the contour lines in this quadrant, the maximum magnitudes of ${\omega ^{{{\mathcal {A}}}}_i\omega ^{{{\mathcal {A}}}}_j{\mathcal {S}}^{{{\mathcal {A}}}}_{ij}}$ are approximately comparable to the maximum magnitudes of${-{\mathcal {S}}^{{{\mathcal {A}}}}_{ij}{\mathcal {S}}^{{{\mathcal {A}}}}_{jk}{\mathcal {S}}^{{{\mathcal {A}}}}_{ki}}$. Near the interface, the shape of the joint PDF shows more strongly compressed and squeezed contour lines indicating that enstrophy production coincides with dissipation production, while enstrophy destruction coincides with dissipation destruction. This behaviour is related to the fact that strain rate and vorticity are simultaneously amplified along the vortex sheets.

### Alignment of vorticity vector and strain-rate eigenvectors

The alignment of ${\varvec{\omega }^{{{\mathcal {A}}}}}$ with the principal strain rate axes directly influences the sign of ${\omega ^{{{\mathcal {A}}}}_i\omega ^{{{\mathcal {A}}}}_j{\mathcal {S}}^{{{\mathcal {A}}}}_{ij}}$. Positive values of ${\omega ^{{{\mathcal {A}}}}_i\omega ^{{{\mathcal {A}}}}_j{\mathcal {S}}^{{{\mathcal {A}}}}_{ij}}$ indicate the production of enstrophy due to the local alignment of vorticity with extensive strain rate eigenvectors, whereas negative values of ${\omega ^{{{\mathcal {A}}}}_i\omega ^{{{\mathcal {A}}}}_j{\mathcal {S}}^{{{\mathcal {A}}}}_{ij}}$ correspond to the attenuation of enstrophy by vortex compression. Numerous studies, using both experiments and DNS, have revealed that the vorticity vector preferentially aligns with the eigenvector corresponding to the intermediate eigenvalue of the strain tensor, this is considered counter-intuitive because the vorticity should preferentially aligns with largest eigenvector. This can be understood by the conservation of angular momentum^37^. It has also been highlighted that the intermediate eigenvalue is predominantly positive^5^

**Figure 15:**
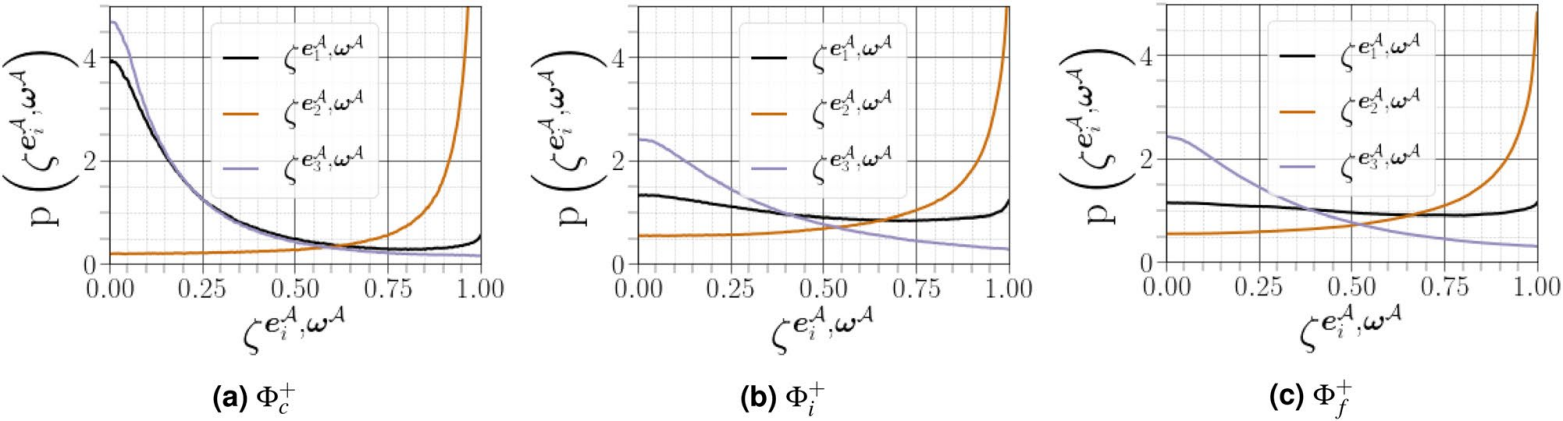
PDFs of alignments between ${\varvec{\omega }^{{{\mathcal {A}}}}}$, and the eigenvectors $\mathbf{e }^{{\mathcal {A}}}_i$, corresponding to the eigenvalues ${\lambda ^{{{\mathcal {A}}}}_i}$ of the strain-rate tensor, conditioned on different distances from the interface.

**Figure 16:**
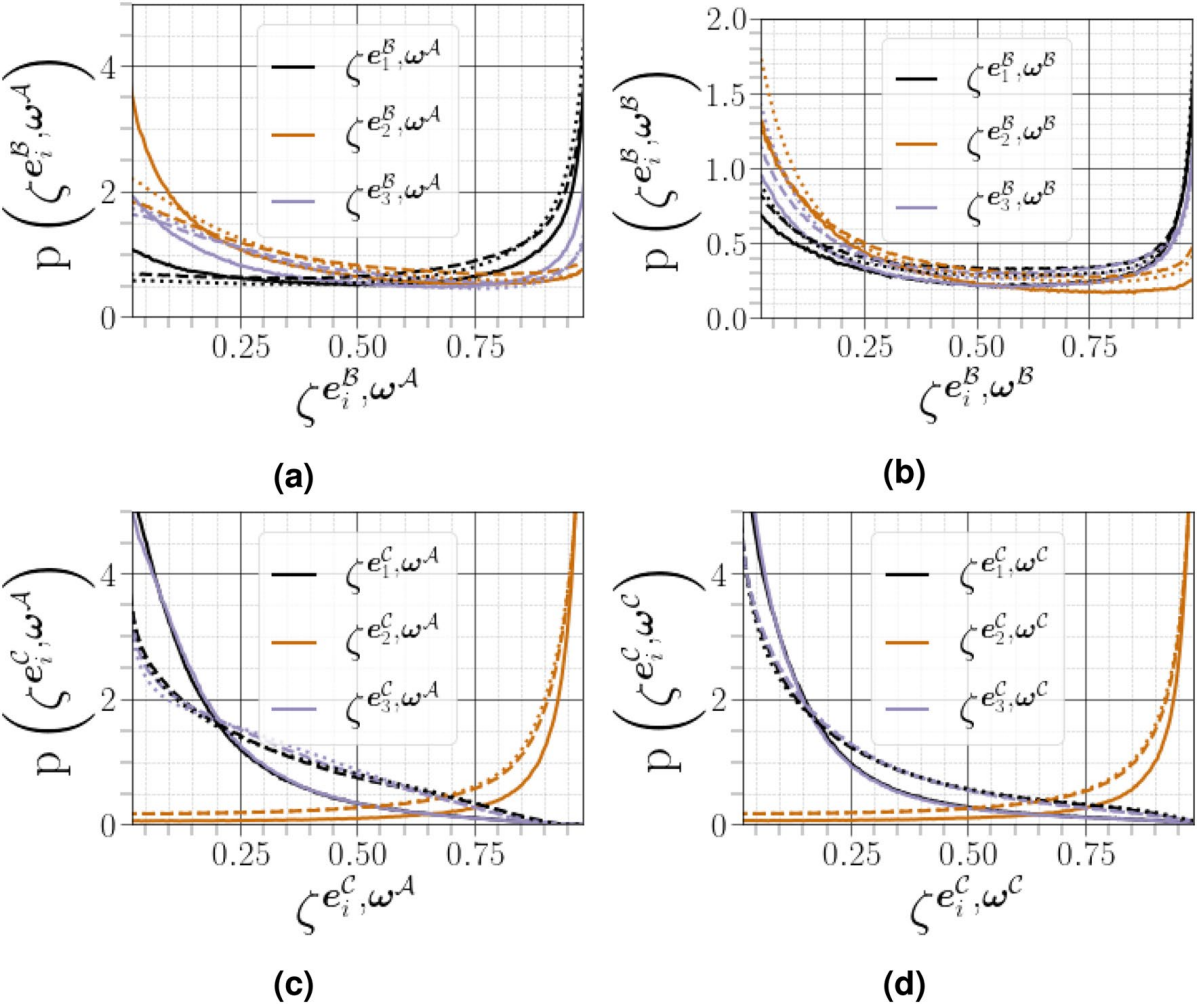
PDFs of the cosines of angles between the vorticity vector and strain eigenvectors for ${{\varvec{\mathcal {A}}}}$, ${{\varvec{\mathcal {B}}}}$ and ${{\varvec{\mathcal {C}}}}$ conditioned on different distances from the interface (solid lines for ${\Phi }^{+}_c$, dashed lines for ${\Phi }^{+}_c$, and dotted lines for ${\Phi }^{+}_f$).

The geometrical analysis far from the carrier-liquid interface reported in Fig. 15 clearly shows that vorticity ${\varvec{\omega }^{{{\mathcal {A}}}}}$ i) preferentially aligns with the intermediate strain direction $\mathbf{e }^{{\mathcal {A}}}_2$, ii) exhibits an arbitrary alignment to the extensive direction $\mathbf{e }^{{\mathcal {A}}}_1$, and iii) tends to be normal to the most compressive principal direction $\mathbf{e }^{{\mathcal {A}}}_3$. The alignment of vorticity with the principal strain direction agrees with the findings of both single phase HIT^38^, and weak shear layers^5^. However, this alignment behaviour changes in premixed flames, especially for small Lewis numbers and high Damköhler numbers^39^. Close to the carrier-liquid interface, we found that i) the parallel alignment of ${\varvec{\omega }^{{{\mathcal {A}}}}}$ with the intermediate strain direction $\mathbf{e }^{{\mathcal {A}}}_2$ becomes more likely, ii) a higher frequency of orthogonality is observed between ${\varvec{\omega }^{{{\mathcal {A}}}}}$ and the compressive strain direction $\mathbf{e }^{{\mathcal {A}}}_3$, and iii) no special orientation of ${\varvec{\omega }^{{{\mathcal {A}}}}}$ and the extensive strain direction does not hold, as evidenced by a strong tendency towards orthogonal alignment between ${\varvec{\omega }^{{{\mathcal {A}}}}}$ and $\mathbf{e }^{{\mathcal {A}}}_1$ with the same magnitude as that obtained between ${\varvec{\omega }^{{{\mathcal {A}}}}}$ and $\mathbf{e }^{{\mathcal {A}}}_3$. A similar change of alignment was observed when approaching the wall in a compressible turbulent boundary layer flow along a flat plate in the absence of either a streamwise mean pressure gradient^40^, or downstream of normal shock wave^29^. This behavior is also obtained under certain conditions in premixed turbulent combustion within the reaction zone^39^. The additive decomposition of ${{\varvec{\mathcal {A}}}}$ into its normal and non-normal parts provides a deeper exploration of previous analysis of turbulence alignment structure, since we can define two additional vorticity vectors ${\varvec{\omega }^{{{\mathcal {B}}}}}$ (for ${{\varvec{\mathcal {B}}}}$) and ${\varvec{\omega }^{{{\mathcal {B}}}}}$ (for ${{\varvec{\mathcal {C}}}}$), and six strain rate eigenvectors ${\mathbf{e }^{{\varvec{\mathcal {B}}}}}$ and ${\mathbf{e }^{{\varvec{\mathcal {C}}}}}$. A subset of these permutations are shown in Fig. 16. Previous results have indicated that vorticity in homogeneous turbulent flows is preferentially aligned to the eigenvector corresponding to the intermediate eigenvalue of the strain tensor ${\varvec{\mathcal {S}}}^{\mathcal {A}}$. Indeed, it has been assumed^41^ that ${\varvec{\omega }^{{{\mathcal {A}}}}}$ stretches in the direction of any eigenvector corresponding to a the strain tensor with a positive eigenvalue, and will eventually become aligned with the eigenvector of the most positive ${\lambda ^{{{\mathcal {A}}}}_1}$. The latter dominant alignment is valid when only the normal contribution of ${{\varvec{\mathcal {A}}}}$ is taken into account, as shown in Fig. 16a. However, a stronger alignment is found between ${{\varvec{\mathcal {A}}}}$ and ${\lambda ^{{{\mathcal {A}}}}_2}$ when considering only the non-normal contribution of ${{\varvec{\mathcal {A}}}}$, with a noticeable anti-alignment between ${\lambda ^{{{\mathcal {A}}}}_1}$ and ${\lambda ^{{{\mathcal {A}}}}_3}$ (see Fig. 16c). This is consistent with the finding of Keylock^13^ for single phase HIT. Multiscale analysis by Leung ^34^, Doan et al.^42^ and Ahmed et al.^43^ also indicate that local effects are responsible for vorticity alignment with the intermediate principal strain rate in wall-bounded turbulent premixed flames. We noted also that i) the alignment between ${\varvec{\omega }^{{{\mathcal {A}}}}}$ and ${\lambda ^{{{\mathcal {A}}}}_2}$ is less strong in the vicinity of the liquid–gas interface, whereas ii) the orthogonality between ${\varvec{\omega }^{{{\mathcal {A}}}}}$ and ${\lambda ^{{{\mathcal {A}}}}_3}$ and ${\lambda ^{{{\mathcal {A}}}}_1}$ is much stronger in this region. The results shown in Fig. 16d and b, *i.e.*, the cosine angle between ${\varvec{\omega }^{{{\mathcal {B}}}}}$ and ${\lambda ^{{{\mathcal {B}}}}_i}$ and between ${\varvec{\omega }^{{{\mathcal {C}}}}}$ and ${\lambda ^{{{\mathcal {C}}}}_i}$, are similar to those shown in Fig. 16a and c.

### Contribution of ${\lambda ^{{{\mathcal {A}}}}_i}$ to enstrophy production and strain self-amplification.

We investigated the statistics of the strain-rate eigenvalues in equations (20) and (21) in order to shed more light on the mechanisms of enstrophy production and strain self-amplification. Table 4 shows the values of ${\left\langle \left( \lambda ^{{{\mathcal {A}}}}_i\right) ^3\right\rangle }$ normalized by ${-\left\langle {\mathcal {S}}^{{{\mathcal {A}}}}_{ij}{\mathcal {S}}^{{{\mathcal {A}}}}_{jk}{\mathcal {S}}^{{{\mathcal {A}}}}_{ki}\right\rangle }$, which represents the positive source term in strain product equation (19). Far from the carrier-liquid interface, there are two extensive strain-rate eigenvalues, which are comparable to both experimental and numerical HIT. This tendency indicates that the positive sign of ${-\left\langle {\mathcal {S}}^{{{\mathcal {A}}}}_{ij}{\mathcal {S}}^{{{\mathcal {A}}}}_{jk}{\mathcal {S}}^{{{\mathcal {A}}}}_{ki}\right\rangle }$ originates mainly from ${-\left\langle \left( \lambda ^{{{\mathcal {A}}}}_3\right) ^3\right\rangle }$. As a consequence, the strain self-amplification process is associated with the stretching and compressing velocity gradients into sheets. When approaching the interface, the values of ${\left\langle \left( \lambda ^{{{\mathcal {A}}}}_i\right) ^3\right\rangle }$ deviate from those reported in HIT simulations and the amplification of sheets seems to result from ${\left\langle \left( \lambda ^{{{\mathcal {A}}}}_1\right) ^3\right\rangle }$.

**Table 4:** Values of ${\left\langle \left( \lambda ^{{{\mathcal {A}}}}_i\right) ^3\right\rangle }$ normalized by the mean strain self-amplification term ${-\left\langle {\mathcal {S}}^{{{\mathcal {A}}}}_{ij}{\mathcal {S}}^{{{\mathcal {A}}}}_{jk}{\mathcal {S}}^{{{\mathcal {A}}}}_{ki}\right\rangle }$ conditioned on different distances from the interface and compared to single phase experimental^44^, and numerical^45^ HIT data.

	${\Phi }_c^{+}$	${\Phi }_i^{+}$	${\Phi }_f^{+}$	Experiments	HIT
${-\left\langle \left( \lambda ^{{{\mathcal {A}}}}_1\right) ^3\right\rangle \Big /\left\langle {\mathcal {S}}^{{{\mathcal {A}}}}_{ij}{\mathcal {S}}^{{{\mathcal {A}}}}_{jk}{\mathcal {S}}^{{{\mathcal {A}}}}_{ki}\right\rangle }$	− 11.06	1.88	0.97	1.62	0.9
${-\left\langle \left( \lambda ^{{{\mathcal {A}}}}_2\right) ^3\right\rangle \Big /\left\langle {\mathcal {S}}^{{{\mathcal {A}}}}_{ij}{\mathcal {S}}^{{{\mathcal {A}}}}_{jk}{\mathcal {S}}^{{{\mathcal {A}}}}_{ki}\right\rangle }$	− 0.01	0.05	0.05	0.05	0.06
${-\left\langle \left( \lambda ^{{{\mathcal {A}}}}_3\right) ^3\right\rangle \Big /\left\langle {\mathcal {S}}^{{{\mathcal {A}}}}_{ij}{\mathcal {S}}^{{{\mathcal {A}}}}_{jk}{\mathcal {S}}^{{{\mathcal {A}}}}_{ki}\right\rangle }$	10.08	− 2.97	− 2.03	− 2.67	− 1.96

**Figure 17:**
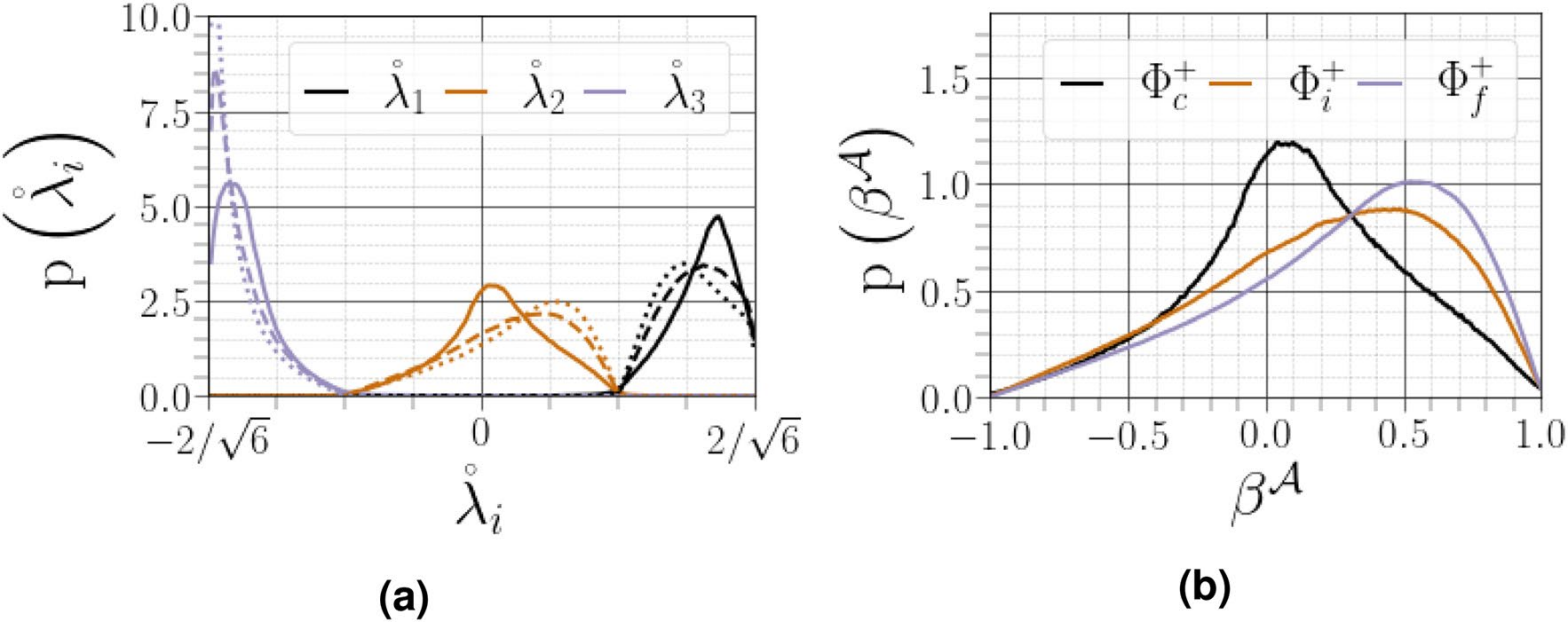
(**a**) PDFs of strain-rate eigenvalues conditioned on different distances from the interface (solid lines for ${\Phi }^{+}_c$, dashed lines for ${\Phi }^{+}_c$ and dotted lines for ${\Phi }^{+}_f$). (**b**) PDFs of $\beta ^{{\mathcal {A}}}$ conditioned on different distances from the interface.

The PDFs of the strain-rate eigenvalues as shown in Fig. 17a. In the vicinity of the carrier-liquid interface, the PDFs of ${\mathring{\lambda }^{\mathcal {A}}_1}$ and ${\mathring{\lambda }^{\mathcal {A}}_3}$ are almost mirror images of the other, while the PDF of the normalized intermediate eigenvalue is symmetric around zero, consistent with increasing two-dimensionality of the flow close to the interface. The PDF of the intermediate strain rate is clearly skewed towards positive values far from the interface, indicating that its behaviour is predominantly driven by vortex stretching and, thus, the amplification of enstrophy. This observation is a characteristic feature of small scale turbulence^38^. To satisfy the incompressibility constraint of the flow, the PDF of ${\lambda ^{{{\mathcal {A}}}}_3}$ exhibits a negatively skewed shape, which breaks its mirror image symmetry with ${\lambda ^{{{\mathcal {A}}}}_1}$. The statistics of ${\lambda ^{{{\mathcal {A}}}}_2}$ were further investigated by plotting the dimensionless quantity ${\beta ^{{\mathcal {A}}}=\sqrt{6}\mathring{\lambda }^{\mathcal {A}}_2}$ in Fig. 17b. Following this normalization proposed by Ashurst et al.^38^, $\beta ^{{\mathcal {A}}}$ should lie within the range $[-1,1]$ owing to the incompressibility constraint, which is evidenced in our present study. The PDF of $\beta ^{{\mathcal {A}}}$ demonstrates, as expected, that i) the values of $\beta ^{{\mathcal {A}}}$ are mostly positive far from the interface, and ii) its right skewness decreases close the interface, becoming more symmetric with respect to zero.

**Figure 18:**
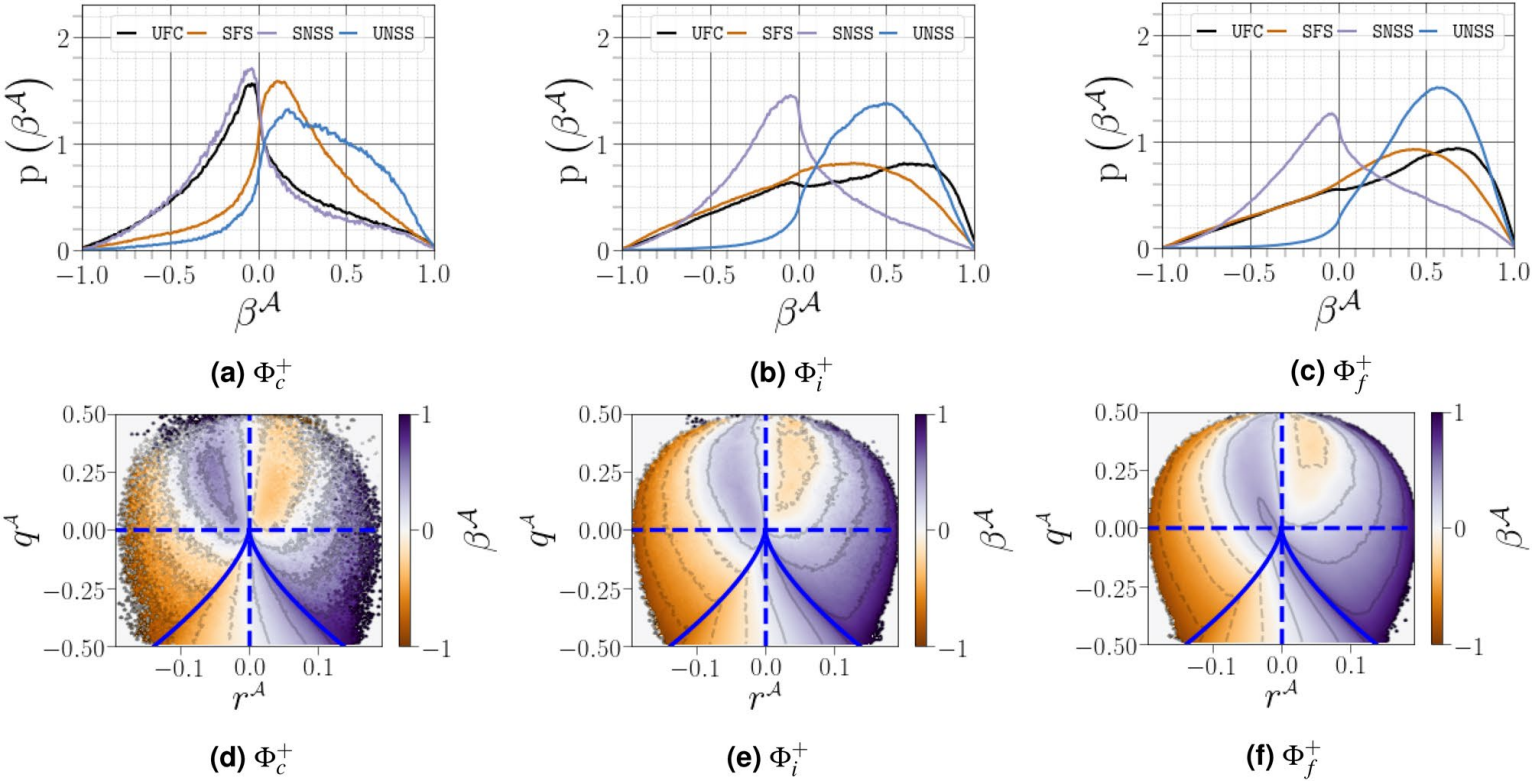
(**a**–**b**–**c**) PDFs of $\beta ^{{\mathcal {A}}}$ conditioned on different distances from the interface as a function of region of the ${(Q^{{{\mathcal {A}}}},R^{{{\mathcal {A}}}})}$ diagram, (**d**–**f**) conditional average of $\beta ^{{\mathcal {A}}}$, $\left\langle \beta ^{{\mathcal {A}}}| q^{{\mathcal {A}}},r^{{\mathcal {A}}}\right\rangle$.

Disaggregating the behaviour of $\beta ^{{\mathcal {A}}}$ in the ${(Q^{{{\mathcal {A}}}},R^{{{\mathcal {A}}}})}$ map shows that, with increasing distance from the interface, $\beta ^{{\mathcal {A}}}$ is strongly positive along the densely populated right discriminant line (UNSS), exhibiting lower dependence on the nodal streamline region of the ${(Q^{{{\mathcal {A}}}},R^{{{\mathcal {A}}}})}$ map (see Fig. 18c). Moreover, the dimensionless values $\beta ^{{\mathcal {A}}}$ are likely to be more expansive in the strain-dominated streamlines (UNSS and $\texttt {SNSS}$) than in the rotation-dominated streamlines (SFS and $\texttt {UFC}$). However, close to the interface (see Fig. 18a and b), the contributions of the nodal streamline region become sufficient to symmetrize the global PDF of $\beta ^{{\mathcal {A}}}$ as shown in Fig. 18c. We also identified a noticeable increase in the occurrence of $\beta ^{{\mathcal {A}}}$ in UFC topology close to the interface.

To obtain a more complete understanding of the distribution of $\beta ^{{\mathcal {A}}}$ in the second and third invariants of ${{\varvec{\mathcal {A}}}}$, we introduced the normalized second and third invariants of the normalized velocity gradient tensor ${b_{ij}={\mathcal {A}}_{ij}/\Vert {\varvec{\mathcal {A}}}\Vert }$ as suggested by Das & Girimaji^46^ as ${q^{{\mathcal {A}}}=Q^{{{\mathcal {A}}}}/\Vert {\varvec{\mathcal {A}}}\Vert ^2,~r^{{\mathcal {A}}}=R^{{{\mathcal {A}}}}/\Vert {\varvec{\mathcal {A}}}\Vert ^3}$. The main difference between the previously presented ${(Q^{{{\mathcal {A}}}},R^{{{\mathcal {A}}}})}$ space of Chong et al.^4^ (featuring normalization by the mean value of the magnitude of vorticity) and the $(q^{{\mathcal {A}}},r^{{\mathcal {A}}})$ space is that the latter provides a mathematically bounded phase plane allowing to deduce, among other features, a streamline shape as observed in ${(Q^{{{\mathcal {A}}}},R^{{{\mathcal {A}}}})}$ map. Indeed, it can be shown^46^ that $-1/2\le q^{{\mathcal {A}}}\le 1/2$ and $-\sqrt{3}/9\le r^{{\mathcal {A}}}\le \sqrt{3}/9$. The lower panel of Fig. 18 shows the conditional normalized mean intermediate strain rate eigenvalue $\beta ^{{\mathcal {A}}}$ in the $(q^{{\mathcal {A}}},r^{{\mathcal {A}}})$ map. We note a close correspondence between the results shown in the upper and lower panels. Moreover, $\beta ^{{\mathcal {A}}}$ seems to show negligible dependence on the second normalized invariant $q^{{\mathcal {A}}}$, exhibiting instead a monotonic increase with respect to the third invariant $q^{{\mathcal {A}}}$ far from the interface (see Fig. 18f). As we approach the interface (see Fig. 18d and e), the probability of being in the first quadrant becomes higher, which explains the symmetrization of the distribution of $\beta ^{{\mathcal {A}}}$ observed in Fig. 18a.

### Statistics of the passive scalar gradient

The scalar dissipation rate $\text {(SDR)}\equiv {\mathcal {N}}_{\varpi }:={\mathcal {D}}\nabla \varpi \cdot \nabla \varpi$ plays a crucial role in determining the rate of micro-mixing and, thus, in the modelling of turbulent reacting flows. The scalar dissipation rate is also used for premixed combustion modelling (see Chakraborty et al.^47^ and references therein). The focus of this section is the turbulence-scalar interaction ${\text {(TSI)}:=-2\rho {\mathcal {D}}\nabla \varpi \cdot {\varvec{\mathcal {S}}}^{\mathcal {A}}\cdot \nabla \varpi }$^48^. This quantity, characterizing the coupling between the velocity and scalar fields in the SDR transport equation, is a leading order source term in the SDR evolution^29^. When expressed in the eigenframe $\mathbf{e }_i^{{\mathcal {A}}}$ of strain rate ${\varvec{\mathcal {S}}}^{\mathcal {A}}$, the TSI term becomes ${-2\rho {\mathcal {D}}\nabla \varpi \cdot {\varvec{\mathcal {S}}}^{\mathcal {A}}\cdot \nabla \varpi =-2\rho {\mathcal {N}}_{\varpi }{\lambda ^{{{\mathcal {A}}}}_i}\cos ^2\left( \mathbf{e }^{{\mathcal {A}}}_i,\mathbf{n }^{\varpi }\right) }$, where $\mathbf{n }^{\varpi }=\nabla \varpi /\Vert \nabla \varpi \Vert$ denotes the normalized scalar gradient. We note that the sign of the $\text {TSI}$ will be dictated by the cosines $|\cos \left( \mathbf{e }^{{\mathcal {A}}}_i,\mathbf{n }^{\varpi }\right) |\equiv \zeta ^{e_i^{{\mathcal {A}}},n^{\varpi }}$ and associated eigenvalues ${{\lambda ^{{{\mathcal {A}}}}_i}}$. Following our discussion of the contribution of ${{\lambda ^{{{\mathcal {A}}}}_i}}$, we here discuss the contribution of $\zeta ^{e_i^{{\mathcal {A}}},n^{\varpi }}$. Geometrical alignment in a single-phase HIT was first reported in Ashurst et al.^38^. It was found that the fluctuating scalar-gradient vector preferentially aligned with the compressive strain-rate eigenvector $e_3^{{\mathcal {A}}}$, which is perpendicular to the intermediate strain-rate eigenvector $e_2^{{\mathcal {A}}}$.

**Figure 19:**
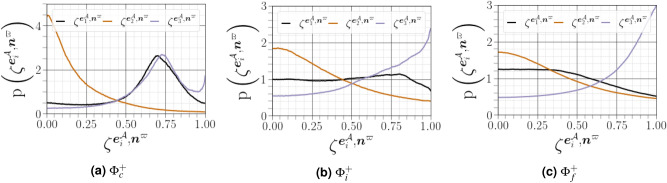
PDFs of alignments between $\mathbf{n }^{\varpi }$, and eigenvectors $\mathbf{e }^{{\mathcal {A}}}_i$, corresponding to the eigenvalues ${\lambda ^{{{\mathcal {A}}}}_i}$ of the strain-rate tensor, conditioned on different distances from the interface.

**Figure 20:**
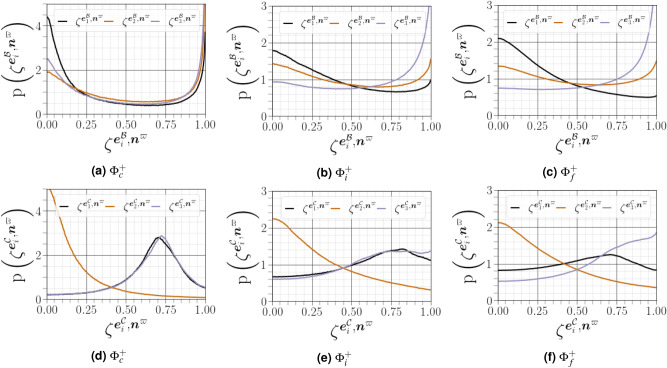
PDFs of alignments between $\mathbf{n }^{\varpi }$, and the strain-rate eigenvectors of tensors ${{\varvec{\mathcal {B}}}}$ and ${{\varvec{\mathcal {C}}}}$ conditioned on different distances from the interface.

In Fig. 19, we show, for the carrier-liquid, changes in alignments between the scalar gradient, $\mathbf{n }^{\varpi }$, and both principal strain directions, by means of space-averaged PDFs of the cosine of the angles between those directions $\zeta ^{e_i^{{\mathcal {A}}},n^{\varpi }}$ at different distances from the interface. The preferred orientation of $\mathbf{n }^{\varpi }$ is reflected along each of the principal strain eigenvectors. Indeed, far from the interface, as inferred from Fig. 19c, the largest contributions to the spatially mean square value of $\mathbf{n }^{\varpi }$ are along $e_3^{{\mathcal {A}}}$, while alignments with the other two strain direction are very similar to the results of Ashurst et al.^38^ for single phase HIT. As the interface is approached, the magnitudes of the alignment change gradually. The orientation of the scalar gradient, lies mostly at about $45^{\circ }$ to both the $e_1^{{\mathcal {A}}}$ and $e_3^{{\mathcal {A}}}$ strain rate directions, suggesting a probable preference to align with the mean strain rate tensor. This alignment close to the interface is compatible with the observations of Abe et al.^49^ near the wall of a turbulent channel flow.

To assess the importance of normal and non-normal effects on the observed alignment in Fig. 19, we disaggregated this alignment between the scalar gradient $n^{\varpi }$ and the eigenvectors $\mathbf{e }^{{\mathcal {B}}}_i$ and $\mathbf{e }^{{\mathcal {C}}}_i$ corresponding to strain-rate tensors of ${{\varvec{\mathcal {B}}}}$ and ${{\varvec{\mathcal {C}}}}$, respectively, as portrayed in Fig. 20. The most striking observation is that the alignments of $\zeta ^{e_i^{{\mathcal {A}}},n^{\varpi }}$ are primarily driven by the non-normal contribution. For the three regions with respect to the liquid-gas interface, the normal effects of ${{\varvec{\mathcal {A}}}}$ modify the magnitude of the alignments, whereas the general alignment $\zeta ^{e_i^{{\mathcal {A}}},n^{\varpi }}$ emerges as a direct consequence of the alignment between the scalar gradient and the eigenvectors of the strain rate of non-normal tensor ${{\varvec{\mathcal {C}}}}$.

**Figure 21:**
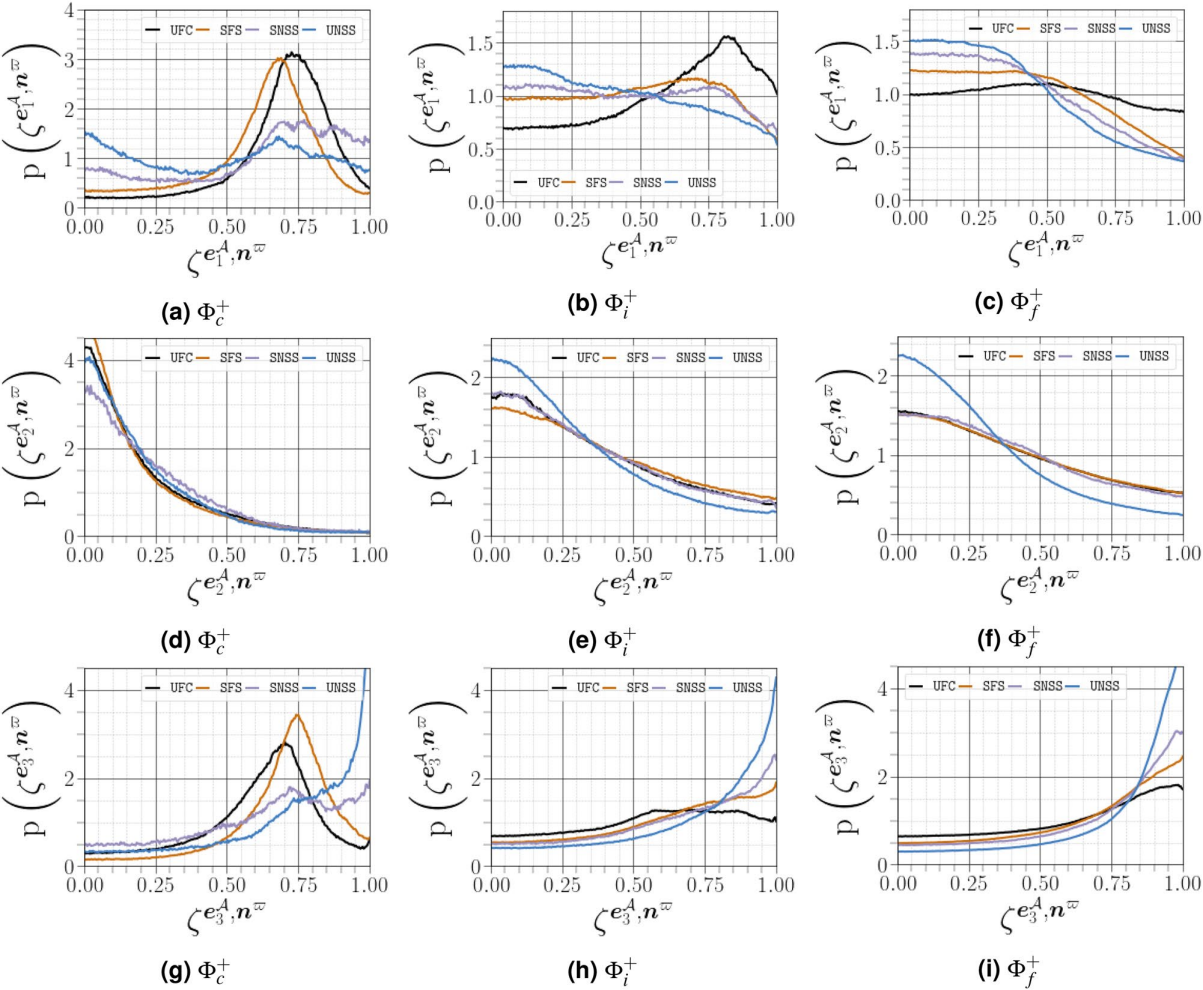
PDFs of $\zeta ^{\mathbf{e }_i^{{\mathcal {A}}},\mathbf{n }^{\varpi }}$ conditioned on different distances from the interface as a function of ${(Q^{{{\mathcal {A}}}},R^{{{\mathcal {A}}}})}$ diagram.

**Figure 22:**
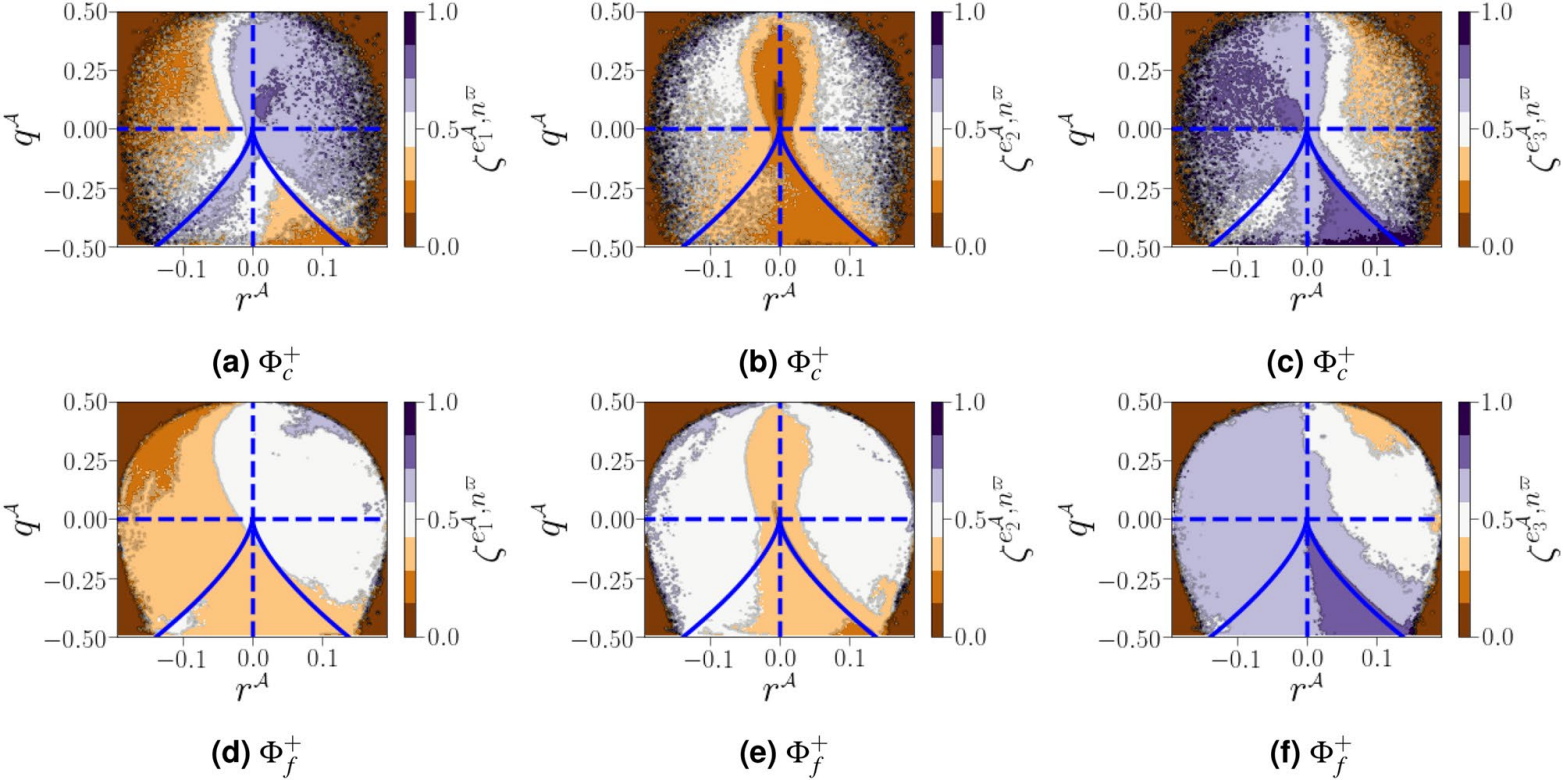
Conditional average $\left\langle \zeta ^{e_i^{{\mathcal {A}}},n^{\varpi }}| q^{{\mathcal {A}}},r^{{\mathcal {A}}}\right\rangle$ at the vicinity of the interface (**a**–**c**) and far from the interface (**d**–**f**).

The PDFs of the alignment between $\mathbf{n }^{\varpi }$ and $\mathbf{e }_i^{{\mathcal {A}}}$ in the four topologies classified in the map ${(Q^{{{\mathcal {A}}}},R^{{{\mathcal {A}}}})}$ are shown in Fig. 21. Preferential alignment of $n^{\varpi }$ with $\mathbf{e }_3^{{\mathcal {A}}}$ was noted for the four topologies independent of their distance from the carrier-liquid interface; these where ordered left to right from most to least aligned as $\texttt {UNSS}>\texttt {SNSS}>\texttt {SFS}>\texttt {UFC}$. This suggests that non-focal strain-dominated topologies enhanced the alignment $\zeta ^{\mathbf{e }_3^{{\mathcal {A}}},\mathbf{n }^{\varpi }}$ to a greater extent than the focal topologies. Moreover, the alignment of $\mathbf{n }^{\varpi }$ to both $e_1^{{\mathcal {A}}}$ and $e_3^{{\mathcal {A}}}$ at about $45^{\circ }$ is much predominant in the focal topologies $\texttt {SFS}$ and $\texttt {UFC}$. Close to the interface, we note that i) the alignment $\zeta ^{\mathbf{e }_1^{{\mathcal {A}}},\mathbf{n }^{\varpi }}$ exhibits a decorrelated trend in non-focal topologies, and ii) $\mathbf{n }^{\varpi }$ is statistically perpendicular to $\mathbf{e }_2^{{\mathcal {A}}}$ in the topologies. The different distributions observed in Fig. 21 were confirmed by plotting the conditional mean $\zeta ^{\mathbf{e }_i^{{\mathcal {A}}},\mathbf{n }^{\varpi }}$ in the $(q^{{\mathcal {A}}},r^{{\mathcal {A}}})$ diagram close and far from the liquid–gas interface (see Fig. 22). Specifically, the strong alignment $\zeta ^{\mathbf{e }_3^{{\mathcal {A}}},\mathbf{n }^{\varpi }}$ in the UNSS is visible far from the interface, and its probability becomes higher in the focal topology close to the interface. Furthermore, the transverse alignment of $\mathbf{n }^{\varpi }$ with $\mathbf{e }_2^{{\mathcal {A}}}$ is mostly concentrated in the negative and positive vicinity of $r^{{\mathcal {A}}}\approx 0$.

## Conclusions

Direct numerical simulation of a forced turbulent two-phase evaporating flow is performed in order to characterize the invariants of the velocity gradient tensor and local flow topology and thus study the dynamics of turbulent scalar interactions. The topological and dissipating behaviour of the flow were investigated for three regions: in the vicinity of liquid pockets, far from these regions, and in an intermediate region as a means of quantifying the transition between the aforementioned regions. Far from the liquid–gas interface, the gaseous flow is almost unaffected by the presence of the liquid phase. Indeed, the joint PDFs of velocity-gradient, rate-of-strain, and rate-of-rotation tensors far from the liquid–gas interface closely resemble canonical and universal single-phase homogeneous isotropic turbulence. However, close to the interface with the liquid-pockets, the flow topology exhibits a boundary-layer-like flow with a predominance of vortex sheets. In the context of LES, these findings suggest viscous regions behaving as turbulent wall-bounded flows, and thus, from the point view of turbulence modelling, a strategy that adapts subgrid modelling to capture droplets interface is plausible. The enstrophy production rate, strain self-amplification and scalar-turbulence interaction were also investigated for topologies classified by the velocity gradient tensor. We found that the mechanisms active in the vicinity of the liquid pockets were different from those acting in the far region; again, we noted a commonality between the present findings and turbulent boundary-layer-like flow. Our results highlight the necessity of considering non-normal effects on the velocity gradient tensor as a means of explaining alignment properties. As demonstrated herein, the application of eigenvalue-focused approaches to turbulence dynamics is not sufficient to understand these mechanisms. It is crucial to consider non-normal effects in conjunction with eigenvalues in order to fully understand the complex dynamics of the velocity gradient tensor.

On another note, the statistics presented in the current analysis, are obtained by conditioning on the distance from the two-phase interface. Similar approaches were adopted in several previous analyses^12,50^. However, the present investigation provides more details on the dynamics of the turbulence-scalar interaction in the context of a two-phase flow, through the introduction of the non-normal effect statistics in the aforementioned fashion, which is, to the best of the authors’ knowledge, have not been considered before. Direct implications of these statistics are discussed in the terms of enstrophy and strain rate generation/dissipation mechanisms which are relevant in the framework of high-fidelity LES model development for multi-phase flows. In this regard, the present analysis points to the importance of considering non-normal effects in future sub-grid scale models to improve their predictive capabilities. Despite the fact that the current analysis is conducted on a canonical configuration, the conclusions made on the basis of small-scale statistics are likely to be valid irrespectively of the flow configuration nature. Nevertheless, conditional statistics on the distance, with respect to the interface, should be considered in a qualitative sense. Indeed, the transition from the interface effect to a standard single-phase behaviour takes place over a distance that may depend on the mean flow nature. 

These present results could be helpful in the construction of reliable SGS models. Typically, the characteristic SGS energy dissipation is an important criterion to assess a model performance. In this respect, the deviatoric part of a new SGS model including the non-local contribution (in the sense of the additional Schur decomposition) could be formulated as

22 $$\begin{aligned} \tau _{ij}^{d}=-2{ \bar{\rho} }\Delta ^2\left( C_{{\mathcal {B}}}\left| \widetilde{\mathcal {\varvec{S}}^{{\mathcal {B}}}}\right| +C_{{\mathcal {C}}}\left| \widetilde{\mathcal {\varvec{S}}^{{\mathcal {C}}}}\right| \right) \left( \widetilde{{\mathcal {S}}_{ij}^{{\mathcal {A}}}}-\frac{1}{3}\delta _{ij}\widetilde{{\mathcal {S}}_{kk}^{{\mathcal {A}}}}\right) , \end{aligned}$$

where the over bar signifies a spatially filtered quantity, and Favre filtered quantity is denoted by a tilde, $C_{{\mathcal {B}}}$ and $C_{{\mathcal {C}}}$ denote the normal coefficient and the non-normal coefficient, respectively, and $\Delta$ is the filter width. The model given by (22) can be downgraded into the standard Smagorinsky model^51^ in the regions where the non-local effects are insignificant. Furthermore, it has the potential to offer a better prediction of the SGS energy dissipation in comparison to the standard approach especially in non-normal dominant regions. An example of such regions, is at the vicinity of the liquid-gas interface where non-local effects dominate.
